# Velocity Prediction of a Pipeline Inspection Gauge (PIG) with Machine Learning

**DOI:** 10.3390/s22239162

**Published:** 2022-11-25

**Authors:** Victor Carvalho Galvão De Freitas, Valbério Gonzaga De Araujo, Daniel Carlos de Carvalho Crisóstomo, Gustavo Fernandes De Lima, Adrião Duarte Dória Neto, Andrés Ortiz Salazar

**Affiliations:** 1 Federal Institute of Education, Science and Technology of Rio Grande do Norte (IFRN), Parnamirim 59143-455, Brazil; 2 Federal Institute of Education, Science and Technology of Rio Grande do Norte (IFRN), Canguaretama 59190-000, Brazil; 3 Department of Sciences and Technology, Federal Rural University of Semi-Arid (DCT-UFERSA), Caraúbas 59780-000, Brazil; 4 Department of Computer Engineering and Automation, Federal University of Rio Grande do Norte (DCA-UFRN), Natal 59072-970, Brazil

**Keywords:** pipeline inspection gauge (PIG), artificial neural networks, embedded systems, raspberry Pi

## Abstract

A device known as a pipeline inspection gauge (PIG) runs through oil and gas pipelines which performs various maintenance operations in the oil and gas industry. The PIG velocity, which plays a role in the efficiency of these operations, is usually determined indirectly from odometers installed in it. Although this is a relatively simple technique, the loss of contact between the odometer wheel and the pipeline results in measurement errors. To help reduce these errors, this investigation employed neural networks to estimate the speed of a prototype PIG, using the pressure difference that acts on the device inside the pipeline and its acceleration instead of using odometers. Static networks (e.g., multilayer perceptron) and recurrent networks (e.g., long short-term memory) were built, and in addition, a prototype PIG was developed with an embedded system based on Raspberry Pi 3 to collect speed, acceleration and pressure data for the model training. The implementation of the supervised neural networks used the Python library TensorFlow package. To train and evaluate the models, we used the PIG testing pipeline facilities available at the Petroleum Evaluation and Measurement Laboratory of the Federal University of Rio Grande do Norte (LAMP/UFRN). The results showed that the models were able to learn the relationship among the differential pressure, acceleration and speed of the PIG. The proposed approach can complement odometer-based systems, increasing the reliability of speed measurements.

## 1. Introduction

Several studies have been undertaken on the topic of the speed control of PIGs. According to [1], a PIG is more effective when it moves at a constant speed. The authors derived mathematical models to analyze the dynamic characteristics in natural gas pipelines, such as the gas flow and the PIG position and velocity. The results included details of the simulation of a velocity excursion event. In a further paper [2], it was stated that PIGs used for batching, cleaning and liquid removal in gas pipelines generally travel along the regular flow of product in the range of 1–5 m/s in liquid pipelines and 2–7 m/s in gas pipelines. For inspection operations, though, the optimal speed range is more narrowly defined (e.g., 0.5–4 m/s for corrosion tools).

In [3], the problem of controlling the speed of a PIG to achieve greater efficiency in cleaning operations was discussed. It was suggested that smart PIGs must move at a constant speed to avoid distortions in the collected data since the sampling time of the acquisition system is constant. The author presented a history of the speed control of PIGs, citing related patents, and described a speed control system that uses a bypass flow valve. The controller regulates a bypass flow based on the feedback of a flowmeter which is controlled by a motorized butterfly valve.

According to the authors of [4], speed control is crucial for different PIG types since the efficiency of cleaning and inspection operations are greatly dependent on the PIG’s speed. They described various speed control methods, classifying them as passive or active. In passive methods, the PIG speed is externally controlled by controlling the pipeline-related variables, such as the operating pressure or flow rate. In active methods, the speed of the PIG inside the pipeline is controlled by internal mechanisms embedded on the device. In [5], a method was proposed for active speed control of PIGs with a brake unit, which is a self-regulated device that generates a drag force that slows down the PIG. The authors proposed a numerical solution for solving the speed-governing equations and simulation results.

In [6,7], some algorithms for neural network techniques for use in PIGs for signal processing to measure pipeline surface corrosion are described that could be used in future research.

In [8], a non-linear sensor fusion algorithm based on an extended Kalman filter (EKF) was used to estimate the trajectory of PIGs. The algorithm combined data from a low-cost IMU (acceleration and angular rate), an odometer (speed), and topographic landmarks (distance). Instead of using an actual PIG moving through a pipeline loop, the authors used an automobile along closed trajectories to perform preliminary experiments. Using only the low-cost IMU, it was not possible to reconstruct the path traveled. However, the performance significantly improved when IMU and additional speed measurements from the odometer and position measurements from the topographic landmarks were combined. In [9], a cleaning PIG with a speed control system was described. Up to three odometers were used to log the distance covered by the PIG and compute the speed for use by the control system (the fastest odometer was automatically chosen). The device also had differential pressure, acceleration and angular rate sensors; however, the authors did not describe what these sensors were used for in this device. According to [10], due to its construction characteristics, odometers are naturally prone to cumulative measurement errors; the main cause of these errors is the pipe welds. In tests performed using an experimental rig, the error varied with the odometer’s speed. The measured distance was greater than the actual distance when the speed was low since the arc length of the weld is longer than the width; the distance was lower when the speed was high due to the loss of contact between the pipe wall and the odometer wheel under this condition. Precisely locating defects that a smart PIG has detected along a pipeline is a significant concern. Hence, tactical-grade inertial measurement units (IMUs) are used to reconstruct the trajectories of the PIG. As stated by [11], these IMUs are accurate but are also large expensive devices, which limits their use in pipelines with diameters below 8" or less. An alternative is to use a micro-electromechanical system (MEMS) IMU, which displays poorer performance but is cheaper and smaller. The authors addressed the issue of aiding a MEMS-based inertial navigation system to replace tactical grade IMUs. They described a new methodology for using MEMS IMUs employing an extended Kalman filter (EKF) and pipeline junctions to increase the position measurement accuracy. In [12], a model that employs neural networks was proposed to determine the relationship between the differential pressure and the speed of a PIG in a testing pipeline. The training set consisted of speed data (calculated from the PIG odometer) and the differential pressure (measured by sensors installed along the pipeline). Upon PIG retrieval, the neural network predicted the speed using data recorded during the run. As reported in [13], experiments were carried out to determine the odometer trajectory on a test bench. A high-speed camera recorded the odometer’s behavior when passing over a weld, enabling detailed analysis of its trajectory. The results showed that changes in the spring force, size, and the material of the odometer could improve accuracy. It was also found that the slower the PIG speed, the greater the accuracy of the odometer. Artificial neural networks (ANNs) have been widely researched and used in the context of systems identification, particularly in the modeling of non-linear systems ([14,15,16]). In [17], it was suggested that neural networks can learn relationships that are difficult to derive from physical modeling. The paper describes the application of neural networks for developing models for predicting process variables, emphasizing recurrent networks in modeling systems that involve temporal relationships.

According to [18], artificial neural networks (ANNs) have valuable properties, such as generalization, robustness, adaptability, intrinsic non-linearity and input-output mapping. These properties make neural networks candidates for solving several problems, such as image-processing, control, identification of dynamic systems and pattern classification. The authors of [19] comprehensively described applications of artificial neural networks in such areas as measurement systems, soft sensors, modeling, fusion, fault diagnosis, and calibration applications. In [20], it was suggested that the use of neural networks is one of the main approaches used to build soft sensors, which are dynamic models devoted to the estimation of plant variables. The author pointed out that neural networks are becoming standard tools for developing non-linear soft sensors due to the good performance obtained for many real-world applications and the availability of software tools that help the designer. In [21], various neural network architectures are described with diverse areas of application, including speech recognition, computer vision, identification and control, medical diagnosis, signal processing, and weather forecasting.

This paper follows previous research undertaken at Universidade Federal do Rio Grande do Norte (UFRN). In [22], pipeline inspection using a device called a pipeline inspection gauge (PIG) was found to be safe and reliable when the PIG operated at low speeds during an inspection. According to [23], pipelines are a key component of an oil and gas supply system, so their maintenance is essential. Among available maintenance techniques, the use of PIGs has been successfully applied in many situations, such as cleaning, product separation and integrity inspection. Finally, the use of neural networks to calculate a PIG’s velocity from the pressure differential was investigated by [12]. The following sections of this article are organized as follows: In Section 2, the research development methodology is described. In Section 3, system implementation is described. In Section 4, the results and discussion are presented. Finally, in Section 5, the conclusions are discussed.

## 2. Research Development Methodology

This section presents some basic concepts and related theoretical issues, including discussion of PIG motion dynamics, inertial sensors, the basics of machine learning and neural networks.

### 2.1. Pipeline Inspection Gauges (PIGs)

**Figure 1:**
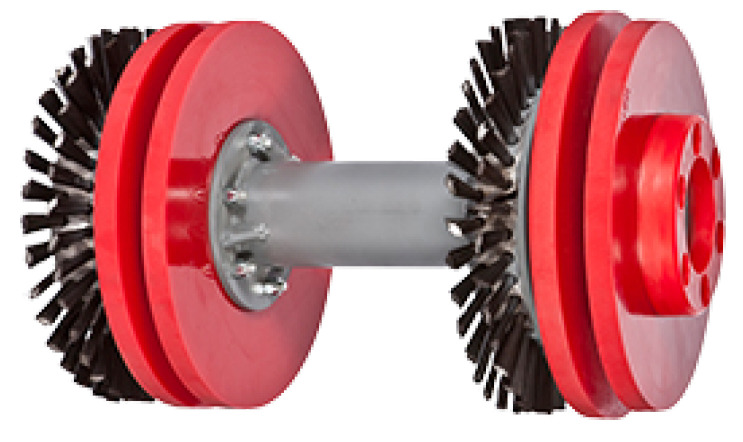
Example of cleaning PIG.

**Figure 2:**
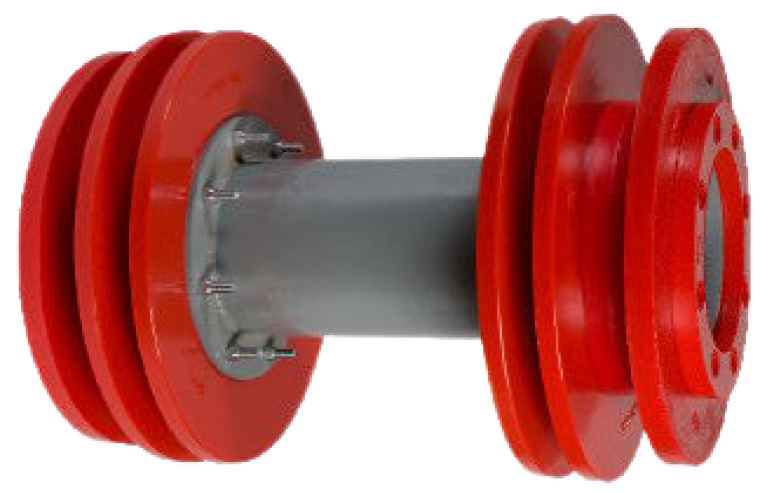
Example of sealing PIG.

**Figure 3:**
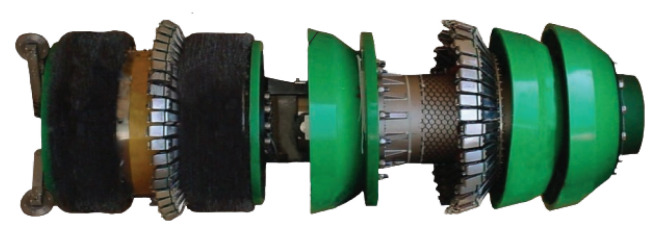
Example of smart PIG.

Pipeline inspection gauges (PIGs) are devices that move inside ducts and are capable of performing a range of tasks, from simple cleaning to detailed inspection of pipeline integrity. Figure 1, Figure 2 and Figure 3 show examples of commercial PIGs designed for different purposes.

### 2.2. PIG Motion Dynamics

**Figure 4:**
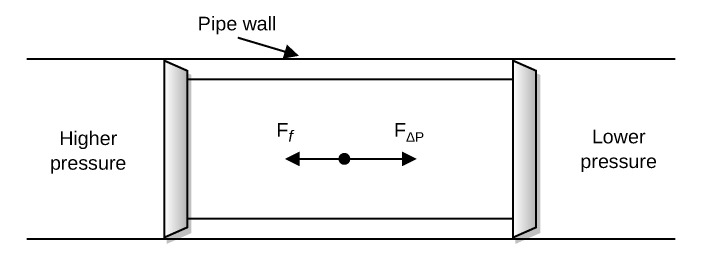
Forces involved in PIG motion.

A PIG travels in the pipeline through the transported fluid due to the differential pressure that acts on the PIG, as illustrated in Figure 4.

Considering a duct parallel to the ground, which implies zero net force on the PIG in the vertical direction, a simplified dynamical model can be obtained from Newton’s Second Law, based on [24], as follows:

(1) $$\begin{array}{ccc} m\frac{dv_{pig}}{dt} & = & F_{\Delta P}-F_{f} \end{array}$$

(2) $$\begin{array}{ccc} F_{\Delta P} & = & A\Delta P \end{array}$$

(3) $$\begin{array}{ccc} F_{f} & = & F_{c}v_{pig} \end{array}$$

where *m* is the mass of the PIG, $v_{pig}$ is the velocity of the PIG, A is the cross-sectional area of the PIG rear, $F_{\Delta P}$ is the driving force, $F_{f}$ represents the friction force, ΔP is the differential pressure that acts on the PIG, and $F_{c}$ is the axial contact force between the PIG and the pipe wall. The dynamic behavior of a PIG inside the pipe is described by its dynamic equation, coupled with the fluid’s governing equations. However, analysis of Equation (3) is sufficient to show that the acceleration of the PIG, and therefore its velocity variation, is directly proportional to the differential pressure. Eventually, some obstacle may impede the PIG motion inside the pipeline, such as debris from the transported product. This situation can lead to the occurrence of so-called velocity excursion, which is explained below.

**Figure 5:**
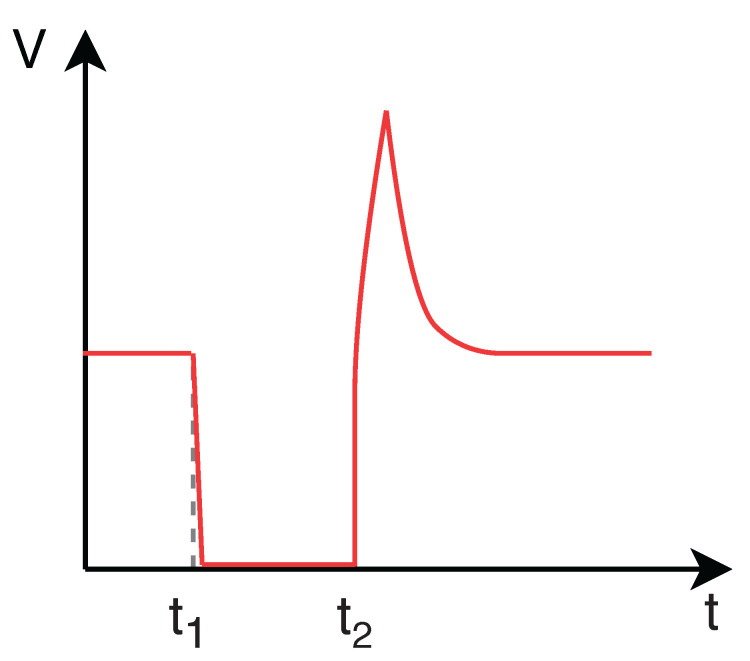
Behavior of velocity (V) and differential pressure (ΔP) with respect to time (t) in the presence of a velocity excursion. PIG velocity.

**Figure 6:**
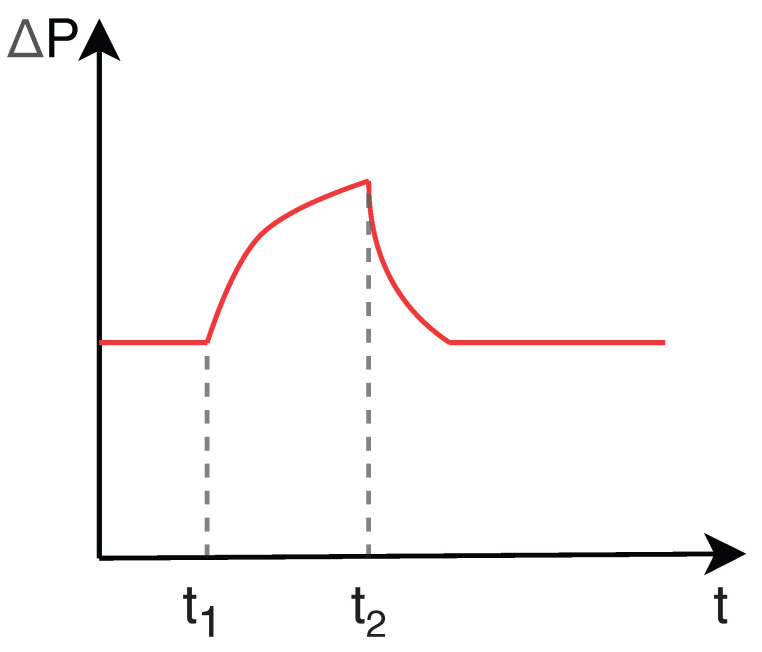
Behavior of velocity (V) and differential pressure (Δ*P*) with respect to time (t) in the presence of a velocity excursion. Differential pressure acting on the PIG.

When an obstacle inside the duct prevents the movement of the PIG, the upstream pressure increases significantly in relation to the downstream pressure. The differential pressure (ΔP) reaches the point where the device can overcome the obstruction, causing a phenomenon known as velocity excursion, which is high velocity (V) reached by the PIG in these conditions. This behavior is illustrated in Figure 5 and Figure 6: the PIG stops at the instant t_1_ and the differential pressure starts increasing from that very moment; at the instant t_2_, as the differential pressure becomes high enough to make the PIG overcome the obstacle, the velocity excursion occurs.

### 2.3. Machine Learning Basics

#### Artificial Intelligence and Machine Learning

Artificial intelligence is a computer science field, born in the 1950s, dedicated to the automation of intellectual tasks usually performed by humans. It encompasses machine learning and deep learning, but also other approaches [25]. Machine learning is a subfield of artificial intelligence that can be defined as the field of study that concerns enabling computers to learn without being explicitly programmed. This definition is credited to the American computer scientist Arthur Samuel. In [26], a more formal definition is provided:

“A computer program is said to learn from experience *E* with respect to some class of tasks *T* and performance measure *P*, if its performance at tasks in *T*, as measured by *P*, improves with experience *E*.”

### 2.4. Artificial Neural Networks

**Figure 7:**
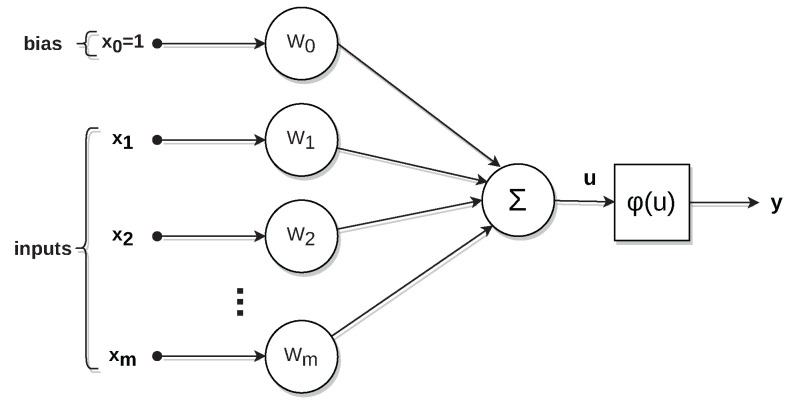
Artificial neuron model.

According to [18], an artificial neural network 1 (ANN) is a distributed system, inspired by the human brain, composed of simple processing units called neurons, which have a natural propensity for acquiring and storing knowledge. Figure 7 shows a neuron model.

#### 2.4.1. Learning Process of Neural Networks

The learning process of a neural network consists of adjusting the synaptic weights and bias of the neurons to minimize a cost function. The algorithm that performs this function is often called the optimizer; the gradient-descent and its variants are amongst the most used optimizers. The ability to learn the behavior of a system from a limited set of samples is one of the main characteristics of neural networks. Once the network has been trained, it is ideally able to produce adequate output from any signals applied to its inputs; that is, it is able to generalize solutions [27].

#### 2.4.2. Architectures of Neural Networks

The architecture of an artificial neural network defines how its neurons are connected. Fundamentally, three distinct neural network architectures can be identified: single-layer feed-forward networks, multilayer feed-forward networks, and recurrent neural networks [18]. In this paper, we employed multilayer feed-forward networks and recurrent networks.

**Figure 8:**
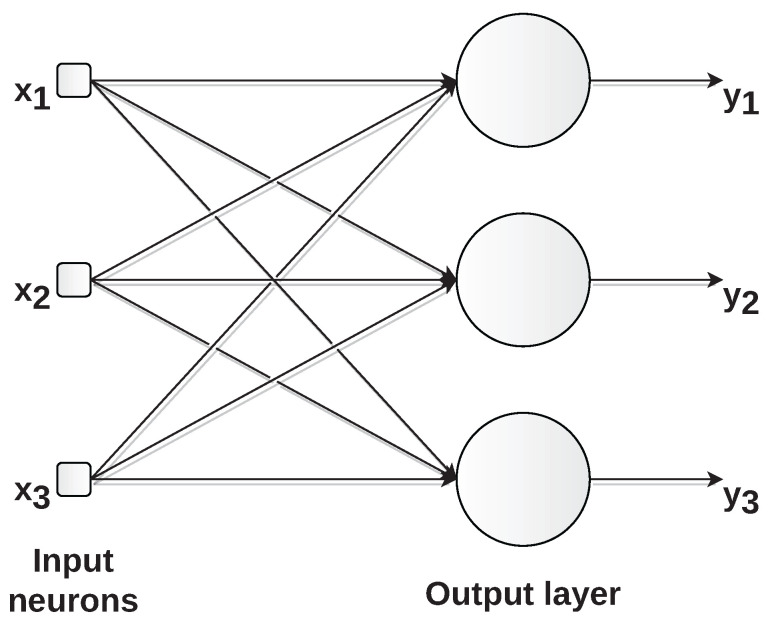
Example of single-layer feed-forward network with three inputs ($x_{1},\;x_{2}$, and $x_{3}$) and three outputs ($y_{1},\;y_{2}$, and $y_{3}$).

In feed-forward networks, neurons are organized in layers and information flows unidirectionally from the input to the network output (hence the term feed-forward). In single-layer feed-forward networks, there is only one layer of neurons, which constitutes the network’s output. We do not count the input layer since no computation is performed there. Figure 8 shows this type of architecture. Neurons are represented by circles; arrows represent the connections between neurons; $x_{1}$, $x_{2}$, and $x_{3}$ are the network inputs; $y_{1}$, $y_{2}$, and $y_{3}$ are the outputs.

**Figure 9:**
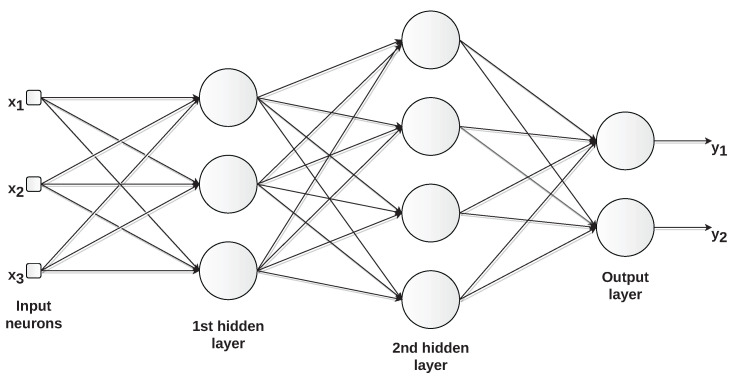
Example of multilayer feed-forward network with three inputs ($x_{1},\;x_{2}$, and $x_{3}$), two hidden layers, and two outputs ($y_{1}$ and $y_{2}$).

In multilayer feed-forward networks, the network comprises one or more hidden layers, whose corresponding neurons are called hidden neurons (Figure 9). They are given this name because the neurons in the hidden layers are not directly visible from either the network’s input or output. A multilayer perceptron (MLP) is a typical multilayer feed-forward network.

**Figure 10:**
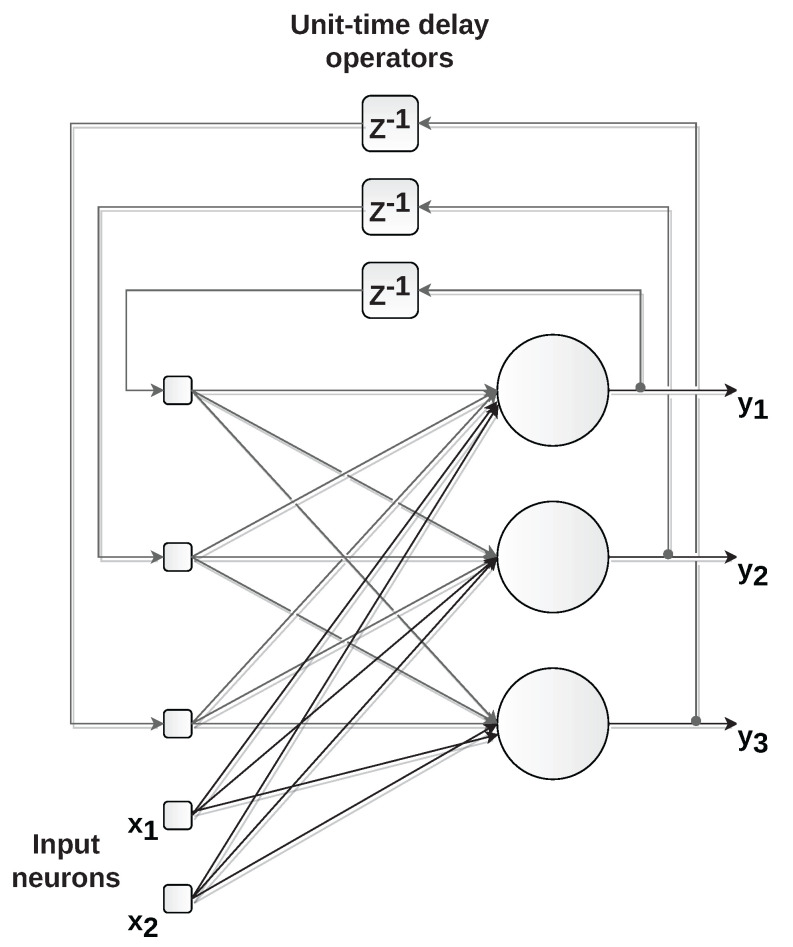
Example of recurrent network with two inputs (x1 and x2) and three outputs (y1,y2, and y3). The time-delayed outputs are used as inputs for feedback on the network.

Networks with recurrent architecture, also known as feedback networks, are made up of neurons whose outputs are used as inputs to the network itself (Figure 10). The introduction of feedback enables recurrent networks to dynamically process information, enabling their use in applications such as time-series forecasting, process control, and systems identification [27]. Examples of recurrent neural networks include non-linear autoregressive networks with exogenous inputs (NARX) and long short-term memory (LSTM).

### 2.5. Artificial Neural Networks and System Identification

The use of mathematical models is inherent in diverse fields of engineering as they are fundamental to better understanding the behavior of a system, in addition to enabling computer simulations. Mathematical modeling can be defined as the area of knowledge that studies techniques for obtaining mathematical models of real systems. A mathematical model is an analog that aims to represent some of the characteristics observed in the real system, for example, its dynamic behavior [28].

There are several techniques for building a mathematical model and even different models for the same system. These techniques are usually grouped according to the following approaches:

- White-box—also known as physical modeling or first-principles modeling—consists of building the model from the analysis of the physical phenomena involved in the system to be modeled;
- Black-box—also called system identification—consists of obtaining the model only from experimental data of the system.
- Gray-box is situated between physical modeling and systems identification and, therefore, combines both information related to the physical phenomena and the experimental data of the system.

One of the most significant benefits of the black-box approach is that only minimal knowledge of the process is required. In contrast, a good understanding of the physical phenomena involved in the process is critical to the development of white-box models [29]. An artificial neural network is essentially a black-box modeling tool, often used to perform non-linear mapping of the input and output of a system.

In the case of so-called dynamic systems, assuming any instant of time, the output depends not only on the present input, but also on its past values [30]. In the following sections, three candidate networks concerning temporal processing, time-delay neural networks (TDNN), non-linear autoregressive networks with exogenous inputs (NARX), and long short-term memory (LSTM) are considered.

#### 2.5.1. Time-Delay Neural Network (TDNN)

**Figure 11:**
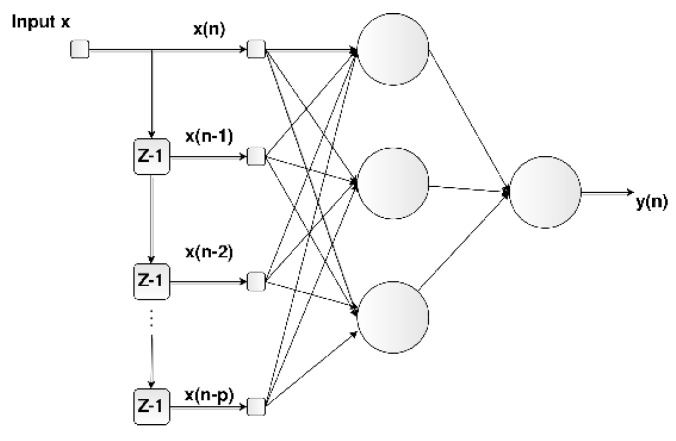
Time-delay neural network (TDNN).

Time may be incorporated into a feed-forward neural network using time-delayed inputs in a structure known as a time-delay neural network (TDNN), as illustrated in Figure 11. A TDNN implements a function given by

(4) $$\begin{array}{ccc} y(n) & = & F[x(n),x(n-1),\cdots ,x(n-p)], \end{array}$$

where *F* is a non-linear function, y(n) is the system’s response, x(n) is the present value of the input signal, and x(n),x(n−1)⋯,x(n−p) are the p past values of the input signal.

#### 2.5.2. Non-Linear Autoregressive Network with Exogenous Inputs (NARX)

**Figure 12:**
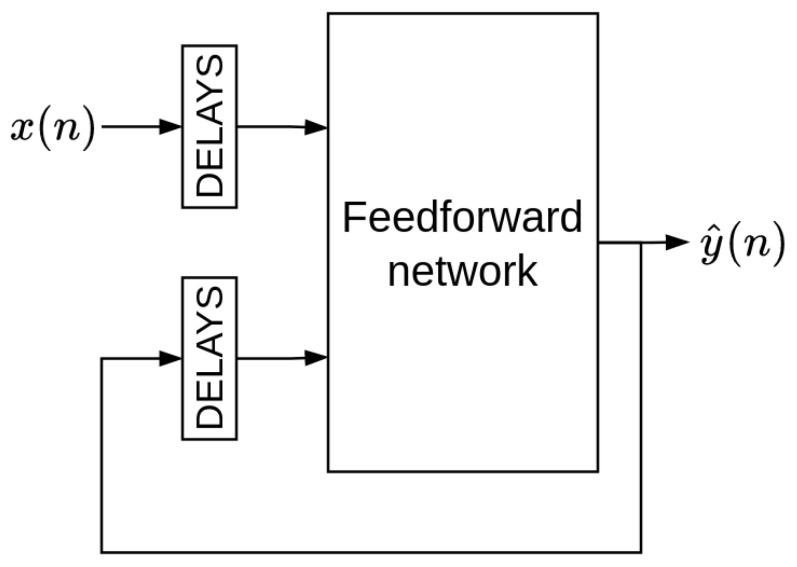
Parallel (closed-loop) configuration.

**Figure 13:**
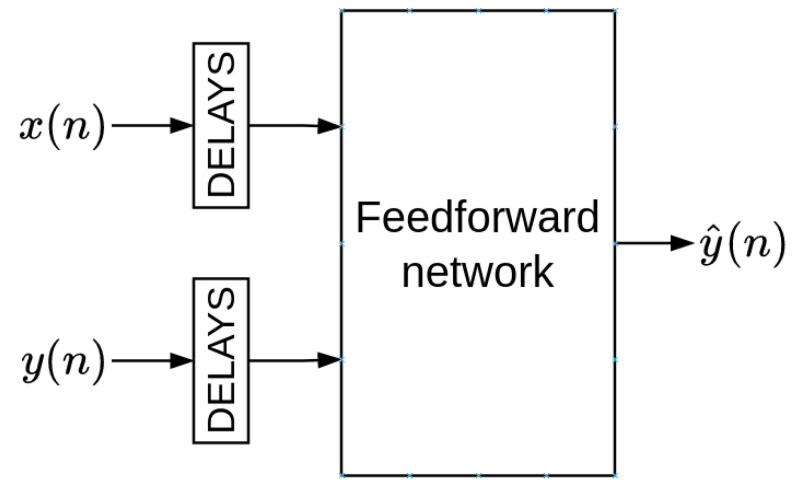
Series-parallel (open-loop) configuration.

Another approach for temporal processing is to incorporate recurrence in a feed-forward neural network using time-delayed inputs and time-delayed outputs. This structure is known as a non-linear autoregressive network with exogenous inputs (NARX). The NARX model can be expressed by

(5) $$\begin{array}{ccc} y(n) & = & F[x(n),x(n-1),\cdots ,x(n-p),y(n-1),\cdots ,y(n-q)], \end{array}$$

where *F* is a non-linear function, y(n) is the system’s response, x(n) is the present value of the input signal, x(n−1),⋯,x(n−p) are the p past values of the input signal, and y(n−1),⋯,y(n−q) are q past outputs. The NARX model can be trained using the parallel (closed-loop) or the series-parallel (open-loop) configuration. In the parallel configuration, the estimated value of the output (target) is fed back into the model (Figure 12). In contrast, in the series-parallel configuration, the true value of the output is used instead of feeding back the estimated output (Figure 13).

#### 2.5.3. Long Short-Term Memory (LSTM)

**Figure 14:**
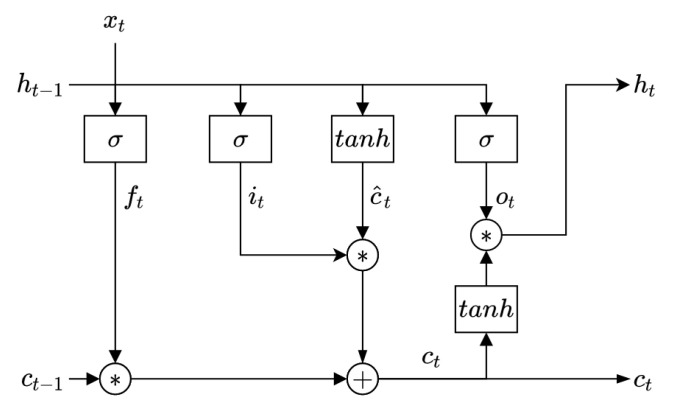
Representation of the LSTM cell.

Long short-term memory (LSTM) is a recurrent neural network first developed by [31]. It can be represented as shown in Figure 14.

where $x_{t}$ is the input vector, $c_{t-1}$ is the previous cell state, $h_{t-1}$ is the previous hidden state, $c_{t}$ is the current cell state, and $h_{t}$ is the current hidden state (output).

LSTM aims to solve the vanishing gradient, gradient explosion, and insufficient long-term ability problems of traditional recurrent neural networks using controllable gates [32]. These gates are the forget gate $f_{t}$, the input gate $i_{t}$, and the output gate $o_{t}$. The LSTM model is described by the following equations:

(6) $$\begin{array}{ccc} f_{t} & = & \sigma (W_{xf}x_{t}+W_{hf}h_{t-1}+b_{f}) \end{array}$$

where σ is the sigmoid function, $x_{t}$ is the input vector, $h_{t-1}$ is the previous hidden state, $W_{xf}$ and $W_{hf}$ are the weight vectors of $x_{t}$ and $h_{t-1}$ on the input gate, respectively; $b_{f}$ is the bias of the input gate.

(7) $$\begin{array}{ccc} i_{t} & = & \sigma (W_{xi}x_{t}+W_{hi}h_{t-1}+b_{i}) \end{array}$$

where $W_{xi}$ and $W_{hi}$ are the weight vectors of $x_{t}$ and $h_{t-1}$ on the input gate, respectively; $b_{i}$ is the bias of the input gate.

(8) $$\begin{array}{ccc} o_{t} & = & \sigma (W_{xo}x_{t}+W_{ho}h_{t-1}+b_{o}) \end{array}$$

where $W_{xo}$ and $W_{ho}$ are the weight vectors of $x_{t}$ and $h_{t-1}$ on the input gate, respectively; $b_{o}$ is the bias of the input gate.

(9) $$\begin{array}{ccc} {\hat{c}}_{t} & = & tanh(W_{xc}x_{t}+W_{hc}h_{t-1}+b_{c}) \end{array}$$

where ${\hat{c}}_{t}$ is an intermediate state, tanh is the hyperbolic tangent function, $W_{xc}$ and $W_{hc}$ are the weight vectors of $x_{t}$ and $h_{t-1}$ on the intermediate state, respectively; $b_{c}$ is the bias of the intermediate state. Finally, the current cell state $c_{t}$ and the current hidden state $h_{t}$ (output) are given by:

(10) $$\begin{array}{ccc} c_{t} & = & f_{t}+c_{t-1}+i_{t}.{\hat{c}}_{t} \end{array}$$

(11) $$\begin{array}{ccc} h_{t} & = & o_{t}.tanh\left(c_{t}\right) \end{array}$$

## 3. System Implementation

This section introduces the materials and methods used to implement the proposed paper. It describes the prototype pipeline inspection gauges (PIGs), the embedded system and sensors, the PIG testing pipeline, the software tools, and data collection and preparation.

**Figure 15:**
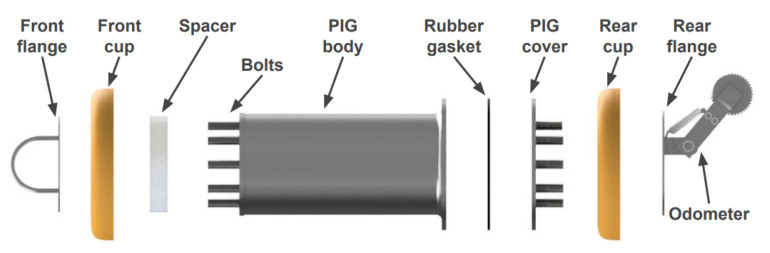
Exploded view of the Prototype PIG 2.

**Figure 16:**
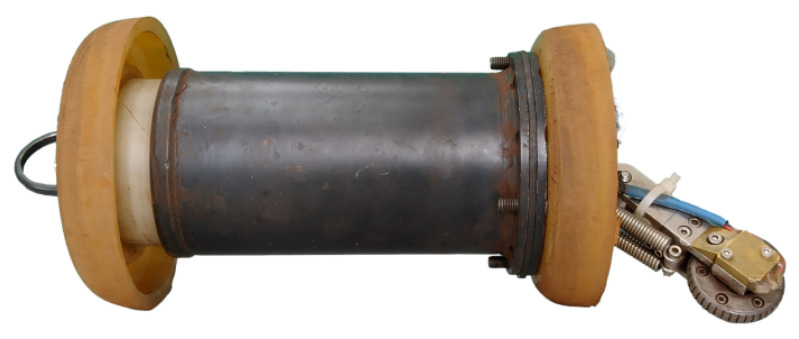
Prototype PIG 2 (side view picture).

**Figure 17:**
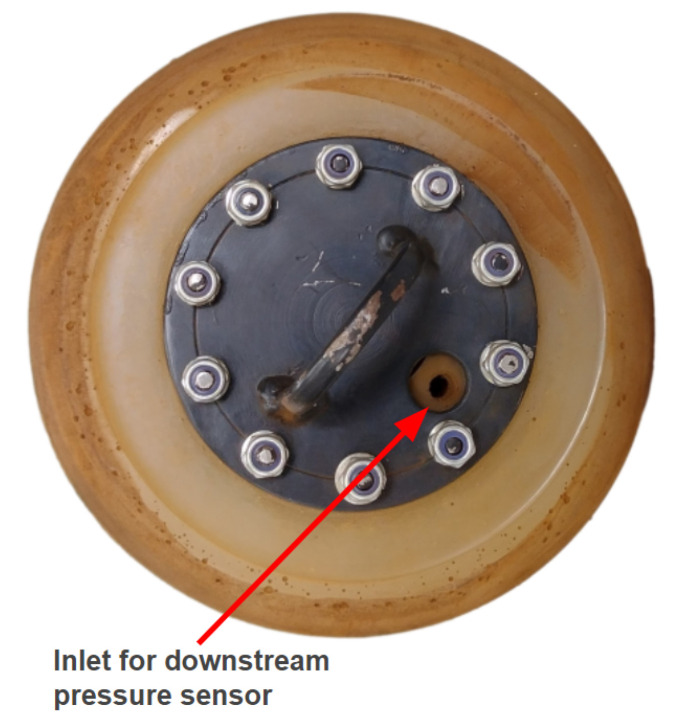
Front and rear views of Prototype PIG 2. Front view.

**Figure 18:**
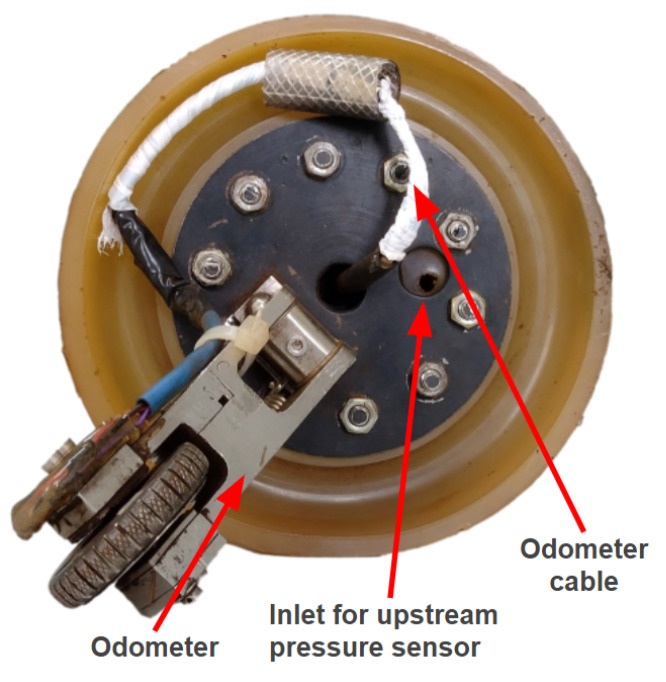
Front and rear views of Prototype PIG 2. Rear view.

The new PIG body has two polyurethane supports (cups) with a diameter of $6^{\prime \prime }$ and a carbon steel body with a diameter of $4.6^{\prime \prime }$ (as in the previous prototype). Figure 15, Figure 16, Figure 17 and Figure 18 show an exploded view of the new PIG, the side view, the front view, and the rear view of the Prototype PIG 2, respectively.

### 3.1. Embedded System and Sensors

**Figure 19:**
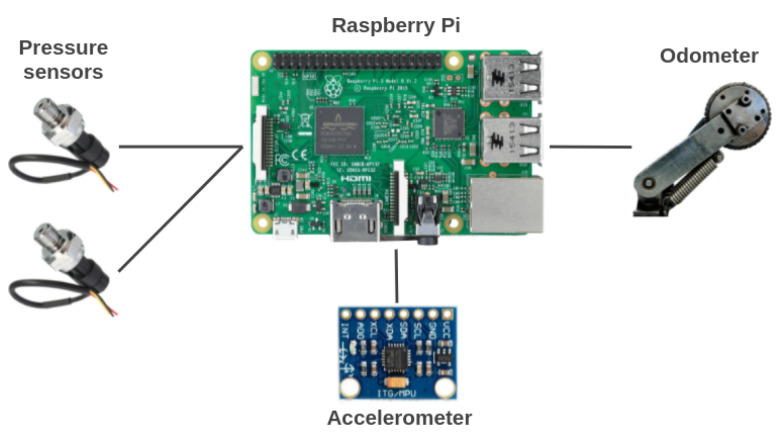
An overall representation of the embedded system’s elements.

The core element of the embedded system is a Raspberry Pi 3 Model B+, as it is used for the acquisition, storage, and processing of the sensors’ data. The sensors measure the following variables: distance, pressure, and acceleration. Figure 19 shows an overall representation of the system.

**Figure 20:**
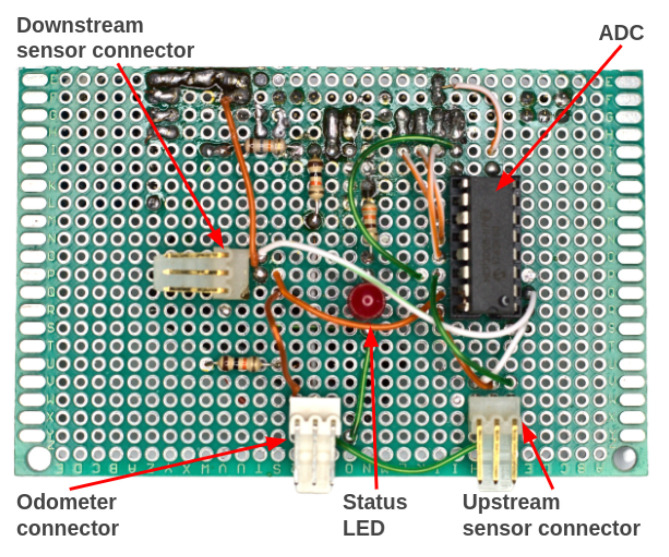
Top view of the Pi Add-On Board. An analog-to-digital converter (ADC) was used to interface the pressure sensors with the Raspberry Pi.

**Figure 21:**
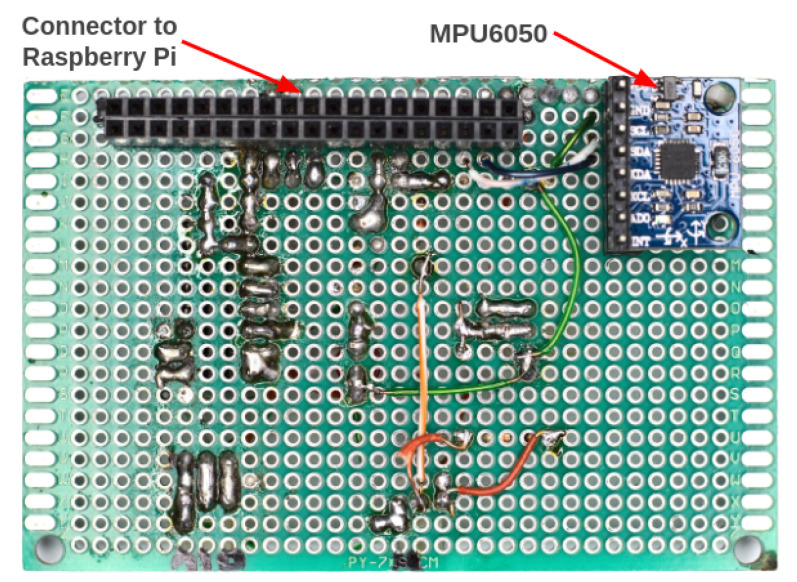
Bottom view of the Pi Add-On Board. The accelerometer (MPU6050) was mounted on the Pi Add-On Board.

Attached on top of the Raspberry Pi is an auxiliary electronic board that we named Pi Add-On Board, which is an auxiliary board constructed from a universal prototype printed-circuit board that we developed to interface the Raspberry Pi with the pressure sensors and the odometer. Figure 20 and Figure 21 show the top and bottom views of the Add-On Board, respectively.

**Figure 22:**
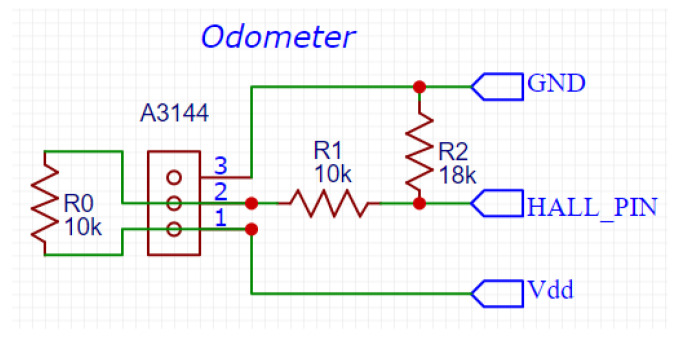
Voltage divider used to reduce the voltage of the odometer’s output signal.

While the Raspberry does not have a built-in analog-to-digital converter (ADC), the pressure sensors’ output is an analog voltage signal, so an external ADC was required to read the pressure from the sensors. The ADC MCP3008 was used, featuring a resolution of 10 bits, 8 input channels, and compatibility with the serial peripheral interface (SPI) protocol. In the odometer case, the output is a digital voltage signal that is either 0 V (low) or 5 V (high). Since the maximum voltage for the Raspberry’s input is 3.3 V, the output of the odometer had to be correctly conditioned, i.e., reduced from 5 V to approximately 3.3 V. This was accomplished using the voltage divider shown in Figure 22. The resistance of R1 and R2 were, respectively, 10 kΩ and 18 kΩ; given a supply voltage of 5 V, this allowed reducing the output voltage to 3.2 V.

In Figure 22, pin 2 is the output of the odometer, HALL_PIN is the input port of the Raspberry Pi that reads the odometer signal, GND is the ground pin, and VDD is the voltage source. The resistor R0 (10 kΩ) between pins 1 and 3 of the odometer connector is required by the A3144 Hall-effect switch.

#### 3.1.1. Raspberry Pi 3 Model B+

The Raspberry Pi is a single-board computer (SBC), a digital computer with all the components necessary for its operation—uch as a microprocessor, input, and output (I/O) interfaces, memory and network interfaces located on a single printed circuit board. Since the launch of the Raspberry Pi in 2012, low-cost SBCs have become quite popular. They have been used for diverse purposes, such as low-cost personal computers, file servers, media centers, the Internet of Things (IoT), robotics, and home automation [33]. Table 1 summarizes some features of the SBC used in this paper, the Raspberry Pi 3 B+.

#### 3.1.2. Pressure Sensors

**Figure 23:**
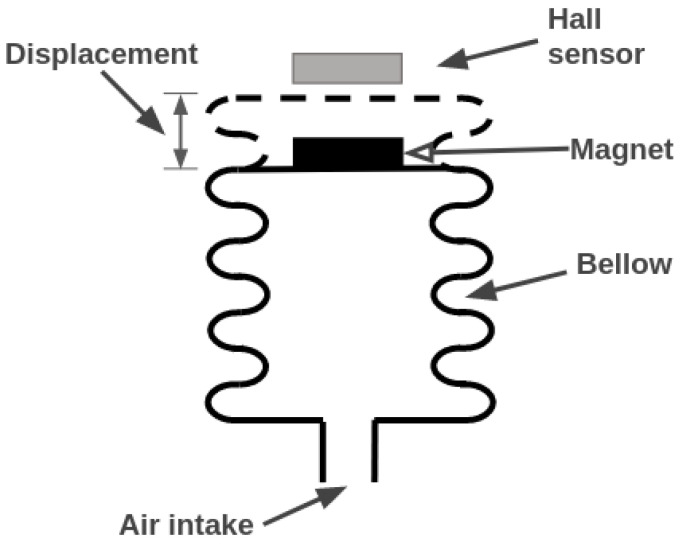
Working principle of the pressure sensor. The Hall-effect sensor is fixed, while the magnet moves according to the applied pressure.

**Figure 24:**
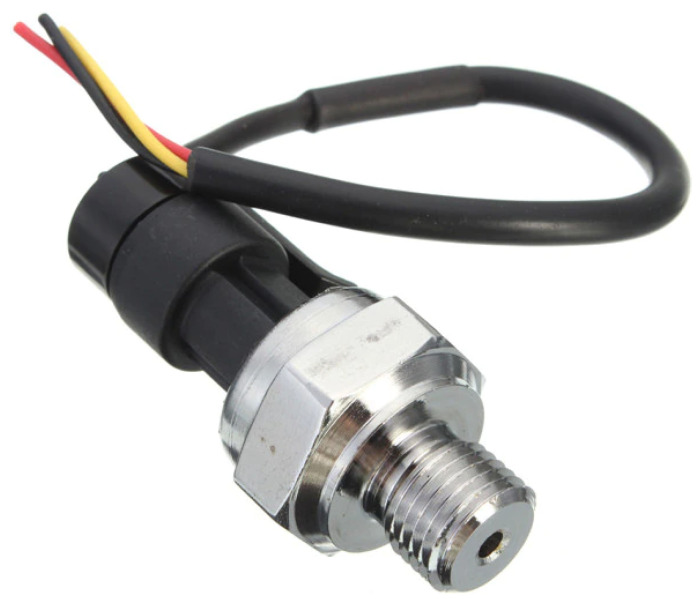
Pressure sensor.

Two pressure sensors were installed on the PIG—one to measure the pressure upstream (behind) and the other to measure the pressure downstream (ahead) of the device. The working principle of the sensors is based on the Hall effect. Figure 23 shows a simplified diagram of the internal construction of the instrument. A bellow with a magnet is placed to move closer to a Hall-effect sensor when the pressure increases since the closer the bellow is to the sensor, the higher the magnetic field. Figure 24 shows the sensor used and Table 2 presents some features of the instrument.

A curve-fitting process was performed to verify the relationship between the pressure and the output voltage of the sensors. This process involves making a certain number of pressure (independent variable) and voltage (dependent variable) observations, then finding a curve that describes the relationship. The voltage of each transducer was measured for six different pressure values (Table 3).

**Figure 25:**
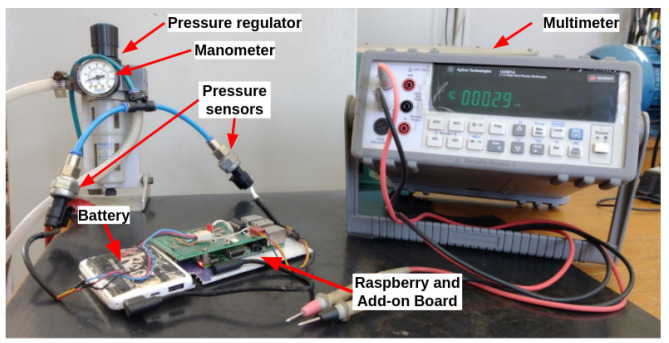
Devices used in the curve-fitting procedure.

Figure 25 shows the devices used to perform the pressure and voltage measurements. The pressure regulator and the manometer were used to control and measure the pressure applied to the sensors, while the multimeter was used to measure the output voltages of the transducers. These voltages were compared with the values provided by the embedded system.

The linear least squares method was used to fit a curve for each sensor from the data presented in Table 3. This method defines the coefficients of a linear model that minimizes the residual sum of squares between the observed data and the data predicted by the linear model. As a result, the following expressions were found:

(12) $$\begin{array}{ccc} P_{UP} & = & 1.241\cdot V_{UP}-0.383 \end{array}$$

(13) $$\begin{array}{ccc} P_{DOWN} & = & 1.242\cdot V_{DOWN}-0.390 \end{array}$$

where $P_{UP}$ is the upstream pressure (bar), $P_{DOWN}$ is the downstream pressure (bar), $V_{UP}$ is the output voltage of the upstream sensor (V), and $V_{DOWN}$ is the output voltage of the downstream sensor (V).

**Figure 26:**
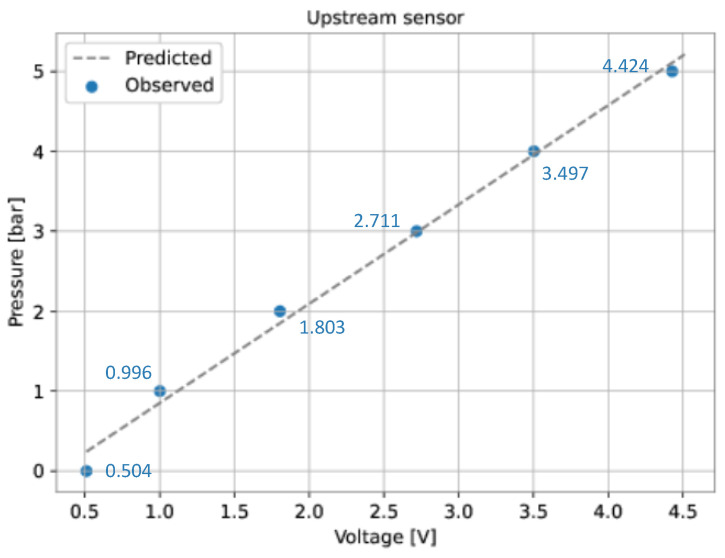
Curve-fitting for the pressure sensors. Upstream sensor.

**Figure 27:**
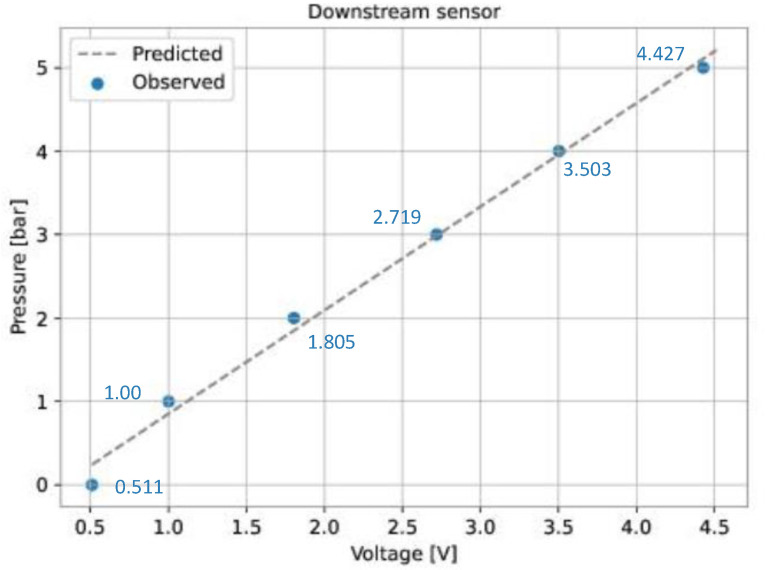
Curve-fitting for the pressure sensors. Downstream sensor.

For each sensor, Figure 26 and Figure 27 show the observed data (Table 3) and the predicted data (Equations (12) and (13)).

#### 3.1.3. Odometer

**Figure 28:**
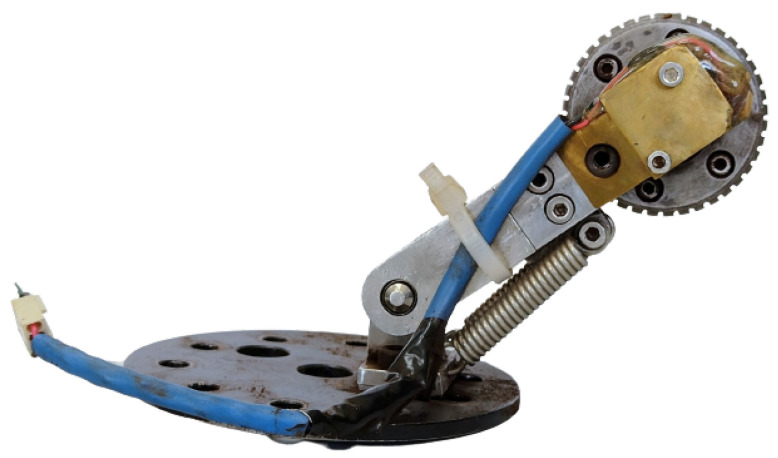
Odometer.

An odometer was attached to the PIG to measure the distance and calculate the speed of the device inside the pipe (Figure 28). The odometer was constructed primarily of stainless steel and was composed of: (a) Base for attaching the odometer to the rear cover of the PIG; (b) An arm that supports the wheel and springs; (c) A wheel with a permanent magnet on its axle; (d) A Hall-effect switch (A3144), placed to detect the magnet; and (e) Two parallel springs to push the wheel against the duct. More details about this odometer can be found at [34].

**Figure 29:**
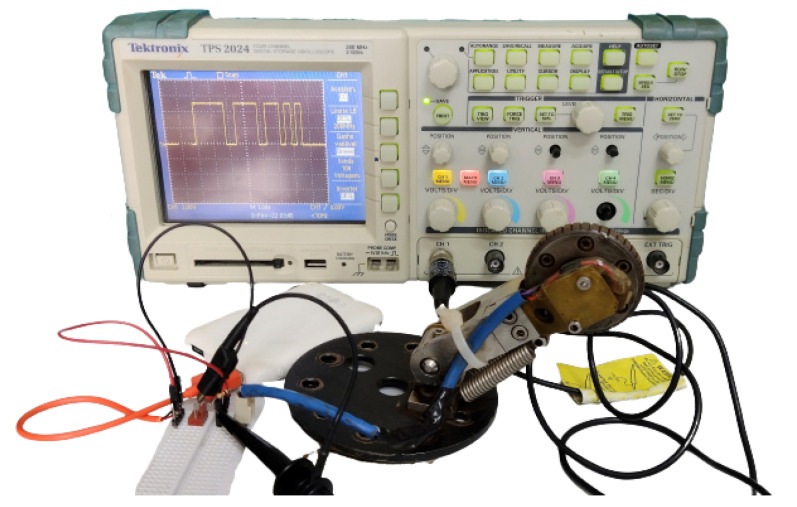
Experimental setup that illustrates the Hall-effect switch’s output.

The Hall-effect switch enables counting of the revolutions of the wheel by detecting the magnet attached to the wheel axle. The output of the switch goes low (0 V) when the field exceeds a certain threshold (the magnet approaches the switch); it goes high (5 V) when the magnetic field is reduced below the threshold (the magnet moves away). Therefore, the output behavior of the switch is a square wave, as observed on the oscilloscope shown in Figure 29. In order to count the rotations of the wheel, the Hall-effect switch was connected to a digital pin of the Raspberry, which generated an interrupt on the rising edge of the square wave.

Considering that the odometer wheel has a perimeter of 0.1539 meters, the following expression was used to measure the distance:

(14) $$\begin{array}{ccc} x[t] & = & 0.1539.n[t] \end{array}$$

where x[t] is the distance travelled between time instants *t* and t−T(m), n[t] is the number of wheel revolutions between *t* and t−T, and *T* is the sampling period. To compute the velocity in the discrete case, an approximation of the derivative of distance with respect to time, a backward Euler differentiator of order one was used:

(15) $$\begin{array}{ccc} v[t] & = & \frac{x[t]-x[t-T]}{T} \end{array}$$

where v[t] is the velocity between time instants *t* and t−T in meters per second (m/s), x[t] is the distance travelled between time instants *t* and t−T, x[t−T] is the distance travelled between time instants *t* and t−T, and *T* is the sampling period.

#### 3.1.4. Accelerometer

**Figure 30:**
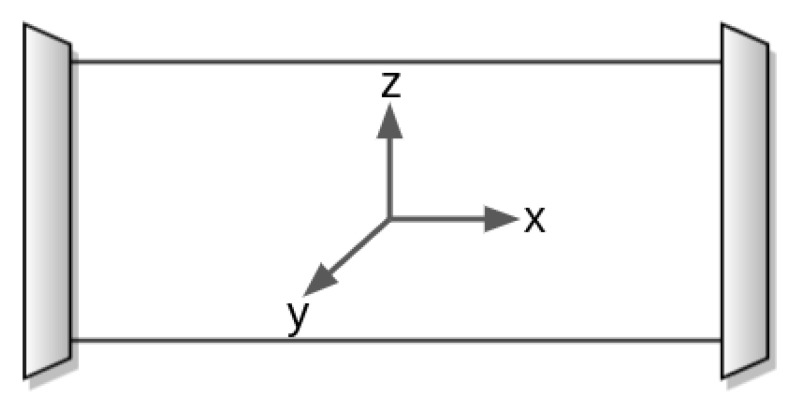
Orientation of the accelerometer inside the PIG.

An accelerometer is used to measure the acceleration of the PIG. The accelerometer is a sensor that detects accelerations by measuring the inertial forces along one, two, or three axes. It can be found in various construction types, including mechanical accelerometers, quartz accelerometers, and micro-electro-mechanical system (MEMS) accelerometers [35]. A MEMS accelerometer employs a proof mass suspended to springs, which displaces in response to an external acceleration. A transducer then detects the displacement. The MPU6050 was configured to measure acceleration between −16 g and 16 g (g = 9.8 m/s^2^). This range was chosen based on values observed in the experimental tests. Inside the PIG, the axes of the accelerometer were oriented as illustrated in Figure 30. The inevitable misalignment between the axes of the accelerometer and the PIG’s axis of motion, and the noise present in the accelerometer’s output signal, made it unfeasible to obtain the velocity from the simple integration of acceleration.

**Figure 31:**
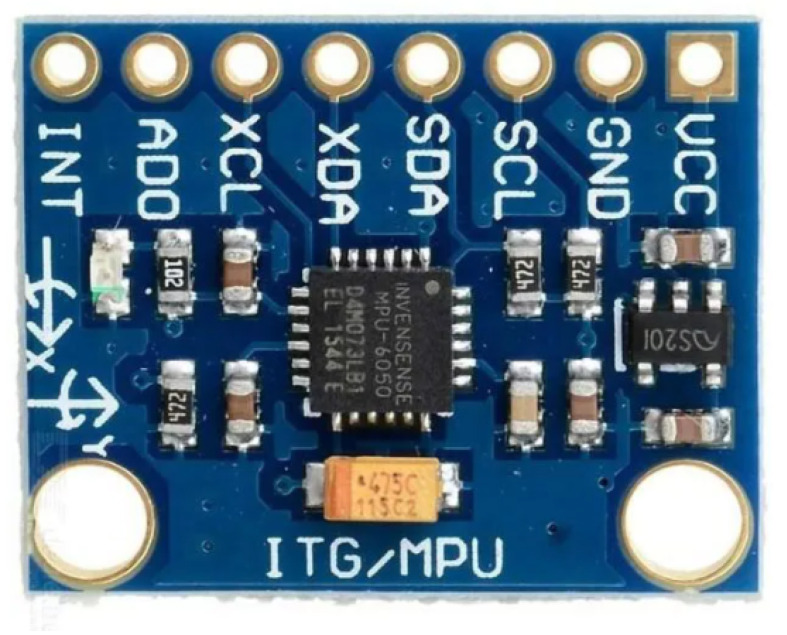
The MEMS accelerometer MPU6050 was used to measure the PIG’s acceleration.

The accelerometer MPU6050 was used (see Figure 31), a MEMS device that combines a 3-axis accelerometer and a 3-axis gyroscope. The MPU6050 uses the inter-integrated circuit (^I2^C) protocol to communicate with the Raspberry Pi.

#### 3.1.5. Power Supply

A portable power bank provided the power supply for the embedded system. A USB to micro-USB cable connected the power bank to the Raspberry Pi micro USB port, then the Raspberry powered the Pi Add-On Board. Table 4 presents the main features of the power bank. To estimate the discharge time of the power supply, we measured the embedded system’s current while simulating typical operating conditions during the PIG run, such as data collection and model inference. The maximum current consumption found for these conditions was 480 mA, which means that, for a power bank capacity of 5000 mAh, the system can work for more than 10 h.

### 3.2. Testing PIG in Pipeline

**Figure 32:**
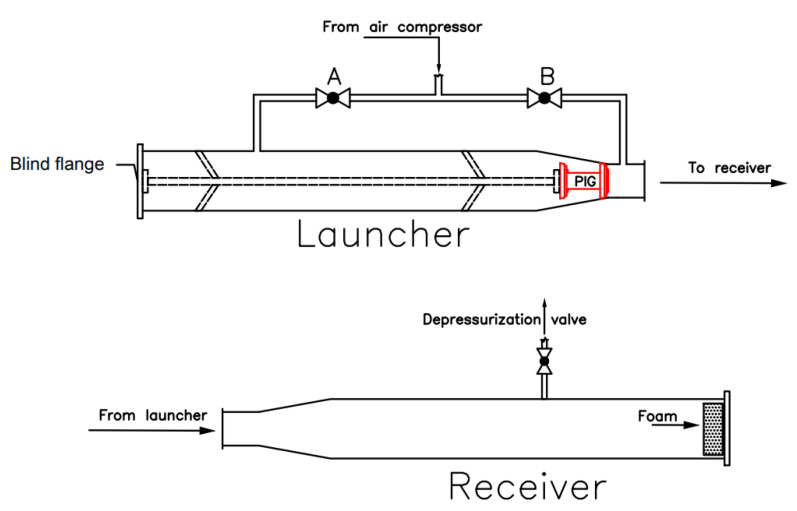
Representation of the PIG launcher and receiver.

In order to obtain the experimental data and train the model, the testing pipeline available at the Petroleum Evaluation and Measurement Laboratory of the Federal University of Rio Grande do Norte (LAMP/UFRN) was used to perform the PIG runs. It has an approximate length of 55 m and a diameter between $6^{\prime \prime }$ and $8^{\prime \prime }$. Blind flanges fixed by screws were installed at the ends of the pipeline. The fluid used was compressed air, whose maximum pressure reached approximately 6 bar. The starting point of a PIG’s run was at the launcher and the endpoint was at the receiver (in Figure 32). To launch the PIG, the launcher was pressurized up to 5 bar and then the receiver was abruptly depressurized causing a differential pressure that pushed the PIG along the pipeline. Finally, the run ended in the receiver, often colliding with a foam placed to absorb the impact.

**Figure 33:**
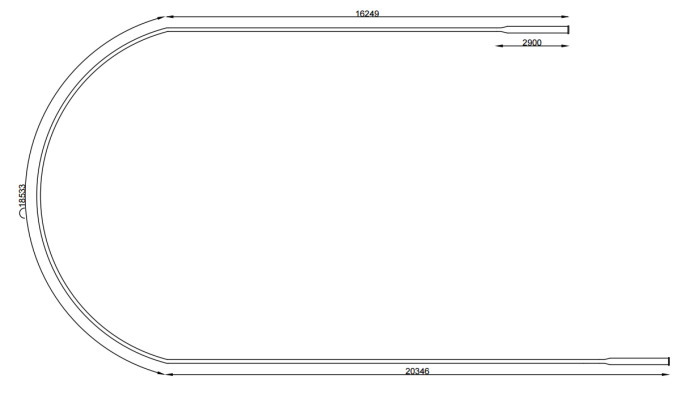
Top-view drawing of the testing pipeline.

**Figure 34:**
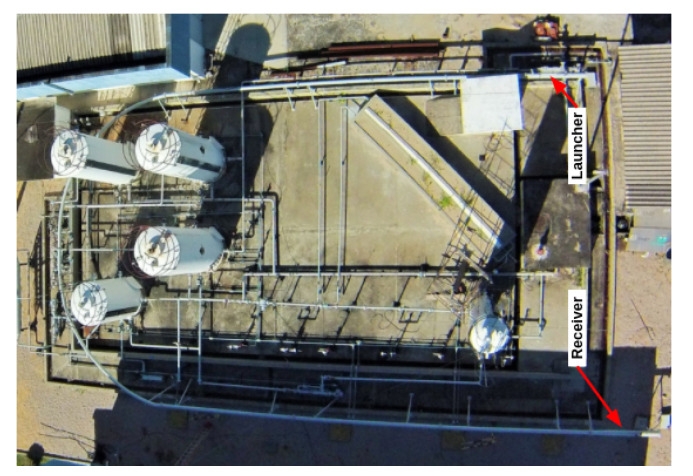
Aerial photo of the testing pipeline.

Figure 33 and Figure 34 show the top-view drawing and an aerial photo of the pipeline. Further details of the development and operation of the pipeline are presented in [23].

### 3.3. Data Collection

**Figure 35:**
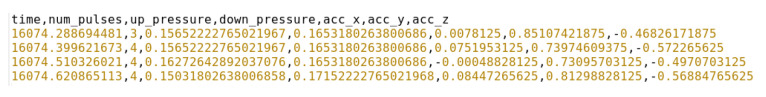
Example of a comma-separated values (CSV) file used to record the data collected from the sensors.

A Python script was developed to measure and record the data from the PIG’s sensors as it travels inside the testing pipeline. The data were recorded in a comma-separated values (CSV) file, as shown in Figure 35.

**Figure 36:**
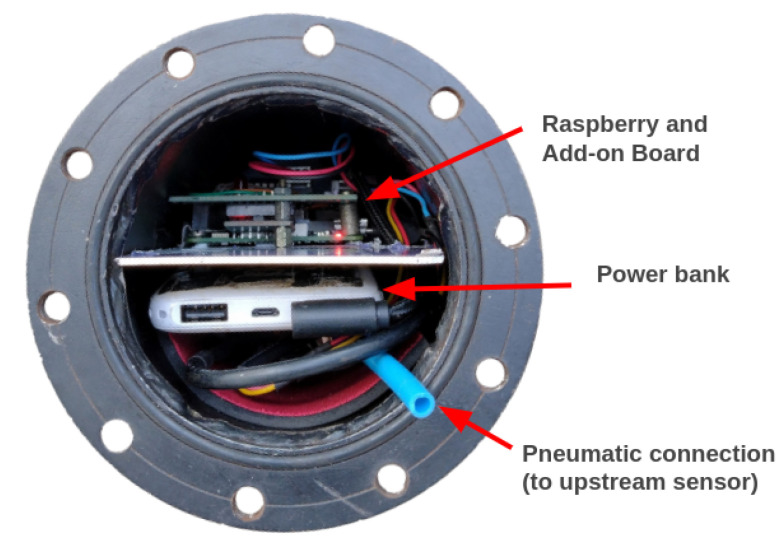
Rear-view of the PIG with the embedded system installed inside.

The column “time” is a timestamp; “num_pulses” is the number of revolutions of the odometer’s wheel, which is proportional to the distance; “up_pressure” and “down_pressure” are the upstream and downstream pressures; $acc_{x}$, $acc_{y}$, and $acc_{z}$ are the accelerations on the x, y, and z axes. The Raspberry Pi was configured to communicate with a laptop computer using Wi-Fi and secure shell (SSH) protocols. This allows the user, for example, to execute commands to run the developed scripts and retrieve data without removing the embedded system from the PIG. Figure 36 shows the embedded system installed inside the PIG.

**Figure 37:**
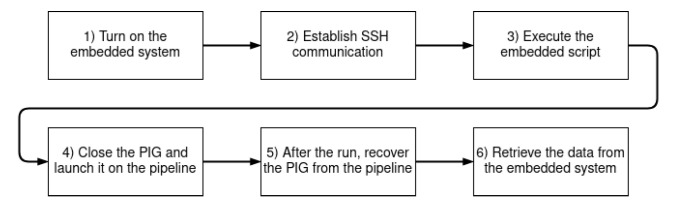
Steps of the data collection procedure.

Figure 37 indicates the steps required for the data collection procedure. First, the electronic devices are connected to the power bank; next, the SSH connection between the laptop and Raspberry Pi is established; using the SSH client on the laptop, the command to execute the data collection script is sent to the Raspberry Pi; once the script starts running, the PIG is closed and inserted into the pipeline; finally, after the PIG is recovered from the pipeline, a command to retrieve the data (i.e., copy the CSV file) from the Raspberry Pi is sent.

### 3.4. Data Preparation

#### 3.4.1. Data Segmentation

**Figure 38:**
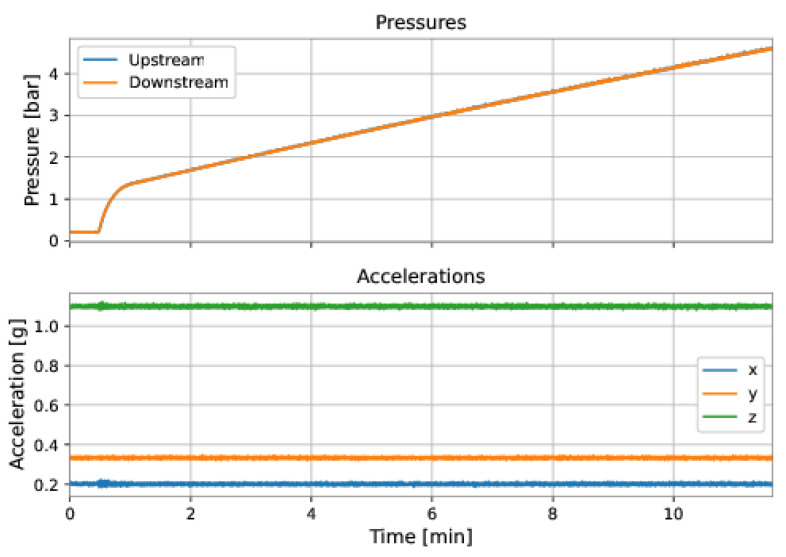
Examples of samples that did not belong to the interest’s regions for the model’s training and, hence, were discarded from the dataset. Initial pressurization of the pipeline.

**Figure 39:**
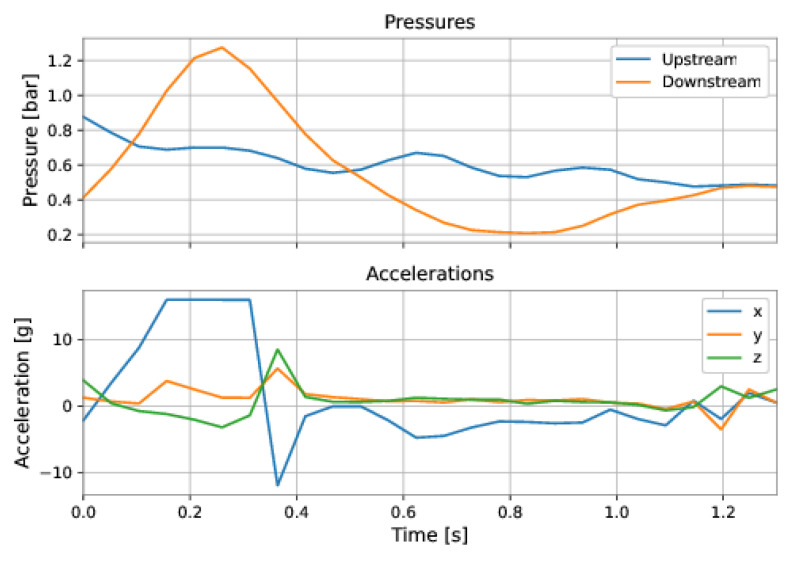
Examples of samples that did not belong to the interest’s regions for the model’s training and, hence, were discarded from the dataset. PIG’s collision at the end of the pipeline.

After the data from a run of the PIG have been retrieved, they were imported into Google Colab for data analysis and preparation. The first step in analyzing a run of PIG was to select the region of interest for the velocity prediction model. It comprised discarding the data corresponding to (a) the pipeline’s initial pressurization, and (b) the collision of PIG with the receiver at the end of the duct. Figure 38 and Figure 39 exemplify these regions.

As shown in Figure 38, the upstream and downstream pressures were equal during the initial pressurization of the pipeline, before the launching of PIG, and the accelerations showed no variation. Figure 39 shows rapid variations in the accelerations and an inversion of the pressures’ signals.

#### 3.4.2. Outliers Treatment

**Figure 40:**
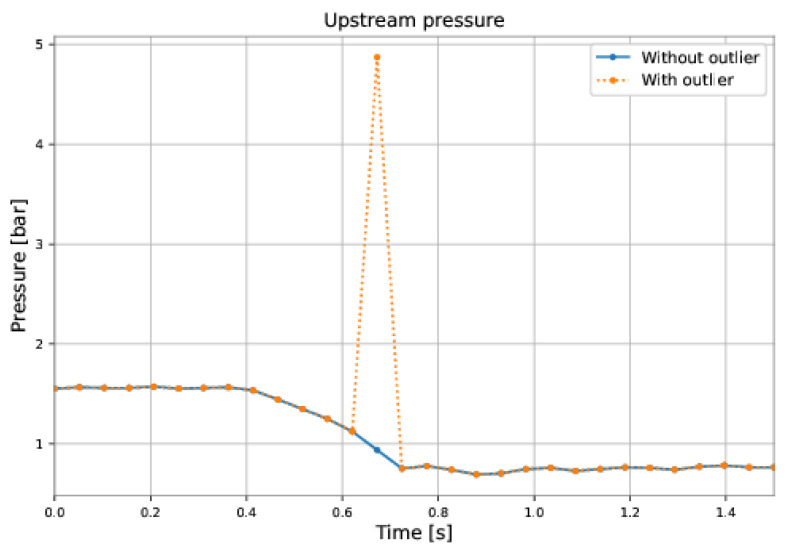
Example of pressure outlier.

The next step was to check for outliers, datapoints that differ significantly from other observations, often due to measurement errors. The outliers were replaced with the average mean of the surrounding values, as shown in Figure 40.

#### 3.4.3. Feature Scaling

We used min-max normalization to make the features lie between 0 and 1. The general formula for min-max normalization in the range [0,1] is given by

(16) $$\begin{array}{ccc} X^{\prime } & = & \frac{X-min(X)}{max(X)-min(X)} \end{array}$$

where $X^{\prime }$ is the vector of the normalized features vector and *X* is the vector of original features.

## 4. Results and Discussion

This section presents the models developed for the PIG velocity prediction of PIG. First, we present the datasets, next, the metric used to evaluate the models, then, the models and their performances and, finally, the results.

### 4.1. Data Sets

Several runs with the PIG were performed, but the data collected in most of the runs presented wrong values of velocity and pressure due to failures of the odometer and malfunctioning of the pressure sensors (due to air leakages into the PIG), respectively. Eventually, it was possible to obtain viable data from two runs.

The first dataset is from a run performed on 15 November 2021 (Run 1), comprising 310 samples and approximately 15 s of the run. The second dataset is from a run performed on 4 March 2022 (Run 2), comprising 373 samples and approximately 18 s of the run. Both datasets were preprocessed according to the data preparation described in the previous Section 3.

In a practical situation, first, a couple of PIG runs would be performed to obtain data to train the model; then the model would be used to predict the velocity of later runs. Aiming to represent this scenario, we used Run 1 to train the model, then Run 2 to test the model. The data collected in each run consisted of the following variables:

- The pressure upstream, i.e., behind the PIG ($P_{up}$);
- The pressure downstream, i.e., in front of the PIG ($P_{down}$);
- The differential pressure (ΔP), defined as
  (17) $$\begin{array}{ccc} \Delta P & = & P_{up}-P_{down}; \end{array}$$
- The acceleration components (acc_x, acc_y, and acc_z) measured by the 3-axes accelerometer;
- The total acceleration (acc_total), defined as acctotal
  (18) $$\begin{array}{ccc} acc\_total & = & \sqrt{acc\_x^{2}+acc\_y^{2}+acc\_z^{2}} \end{array}$$
- The velocity of the PIG is calculated from the odometer measurements.

**Figure 41:**
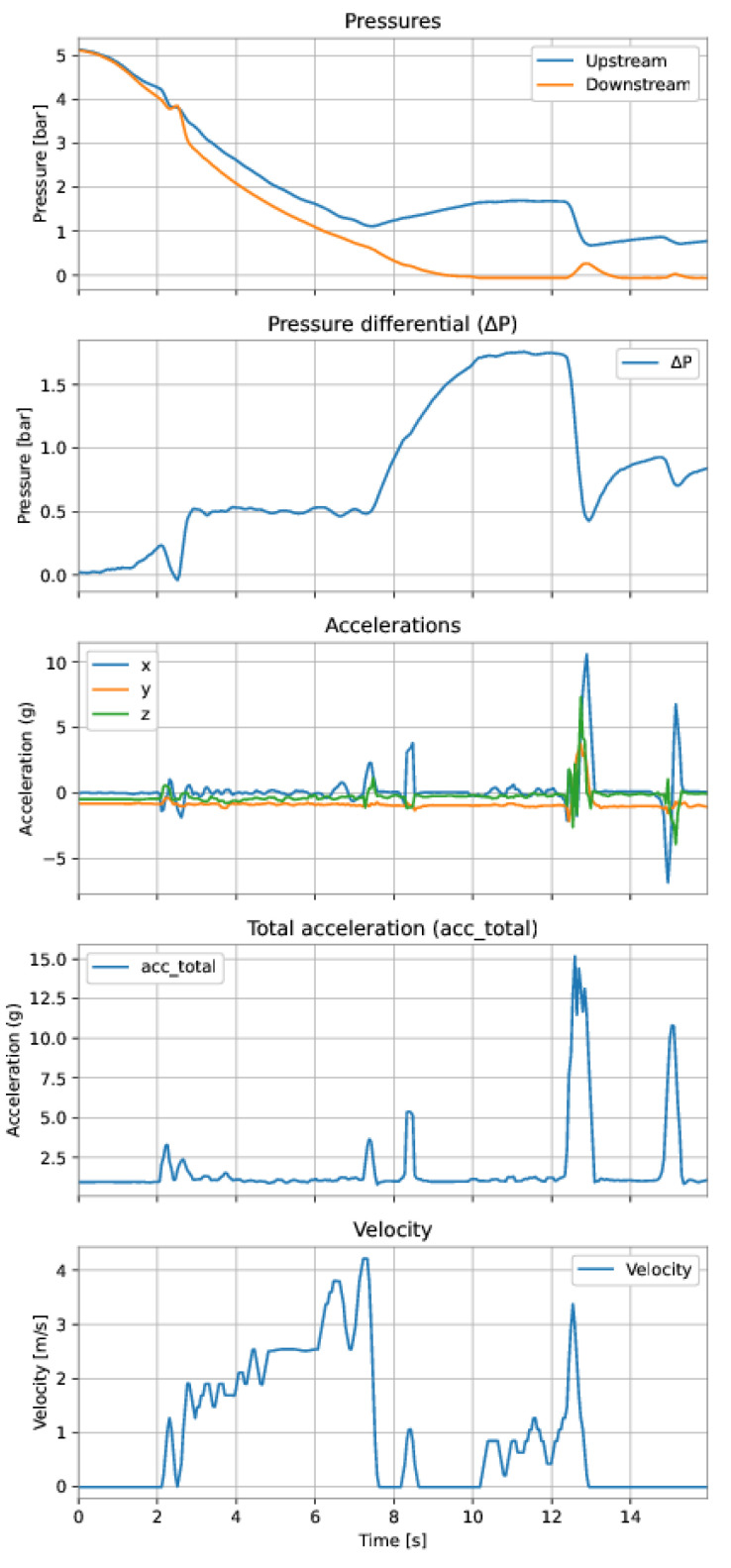
Training dataset. After instant 14 s, it is possible to see a probable inconsistency in the velocity measurement, since the differential pressure and the accelerations varied significantly while the velocity remained z.

**Figure 42:**
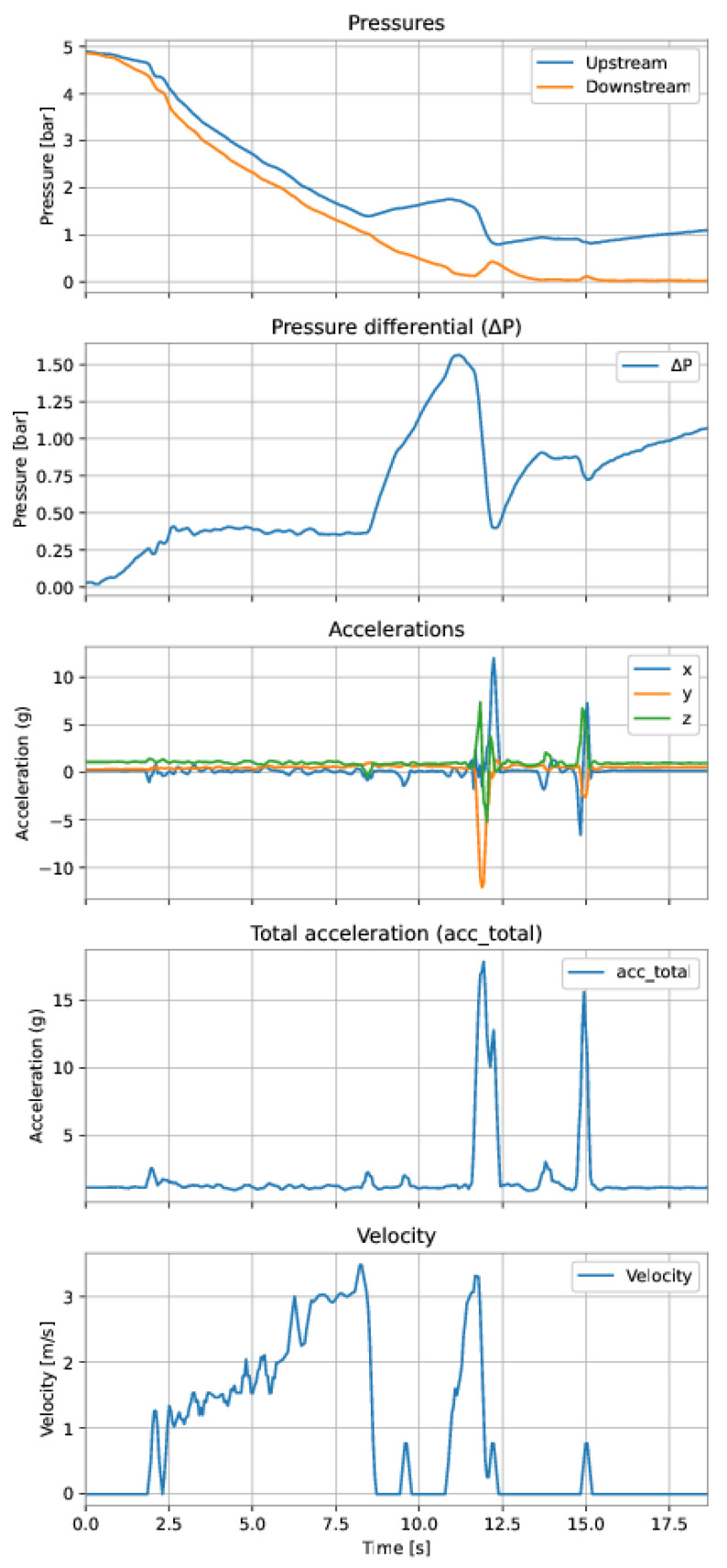
Test dataset.

The variables were either measured (upstream pressure, downstream pressure, and accelerations on the three axes) or calculated (differential pressure, total acceleration, and velocity). The model’s target (output variable) was the PIG’s velocity and the features (input variables) were defined for each model. Figure 41 and Figure 42 show the training data (Run 1) and the test data (Run 2), respectively.

### 4.2. Model Evaluation

The models were evaluated with the root mean square error (RMSE), which is given by

(19) $$\begin{array}{ccc} RMSE & = & \sqrt{\frac{1}{N}\sum_{i=1}^{N}{\left(y_{i}{\hat{y}}_{i}\right)}^{2}} \end{array}$$

where *N* is the number of samples, $y_{i}$ is the true value of the i-th sample, and ${\hat{y}}_{i}$ is the predicted value of the i-th sample.

The RMSE measures the prediction error, determining the overall deviation between estimated and actual values. It is a widely used metric for evaluating the performance of regression models. The lower the RMSE, the better the performance of the model. RMSE was presented on the training and test sets for the models developed. The best performance on the test set was the main criterion we used to define the best model.

### 4.3. PIG Velocity Prediction Models

Before employing more complex models to predict the PIG velocity, a multivariate linear regression technique based on ordinary least-squares available in the Python library Scikit-learn [36] was used as a baseline model. A baseline model is helpful to evaluate if a simple model, such as linear regression, can estimate the PIG velocity, or if more complex models are required (such as artificial neural networks).

**Figure 43:**
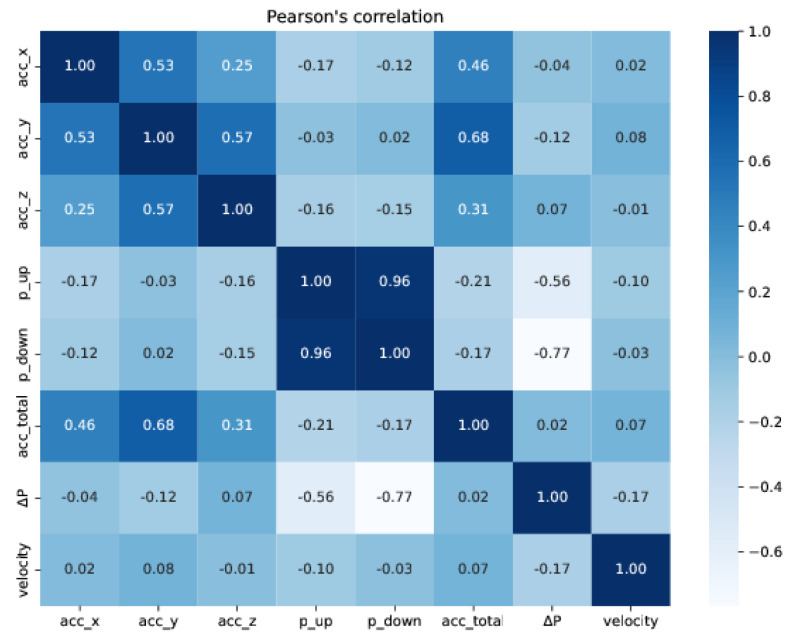
Heat map representation of Pearson’s correlations for the training set.

**Figure 44:**
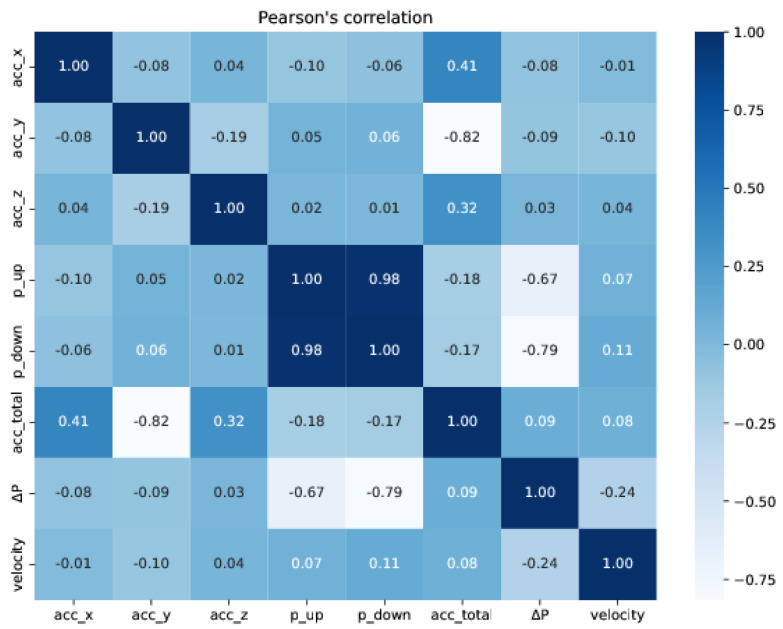
Heat map representation of Pearson’s correlations for the test set.

We first computed the Pearson’s correlation coefficient to evaluate the linear correlation between the variables of the training data: acc_x, acc_y, and acc_z (accelerations on x, y, and z axes); p_up and p_down (upstream and downstream pressures); acc_total (total acceleration), ΔP (differential pressure), and velocity. Figure 43 and Figure 44 show the correlations on the training and test sets, respectively, using a heat map representation. According to the correlation heat maps, the velocity has no strong linear correlation with any of the input features, suggesting that the linear regression model might not be an adequate candidate to predict the velocity of the PIG. Table 5 shows the training and test losses obtained by the models. Each model corresponds to a different combination of inputs.

**Figure 45:**
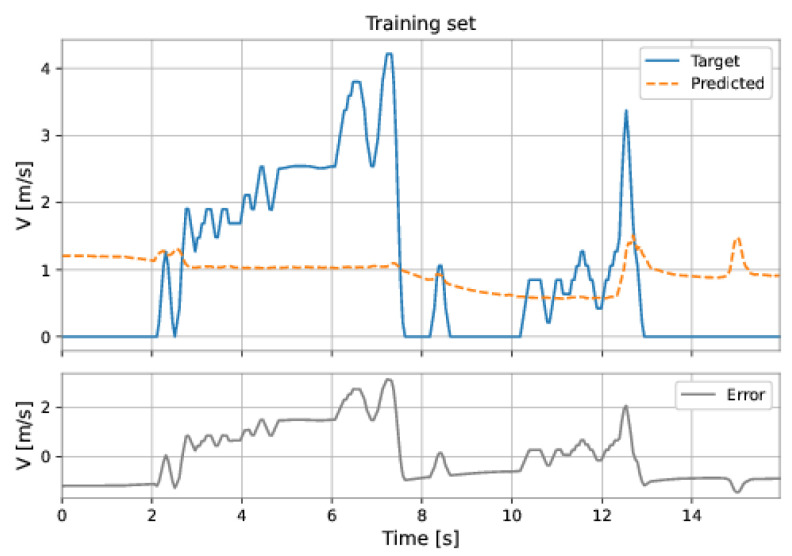
Linear regression predictions on the training. The orange dashed line is the velocity predicted by the model, the blue solid line is the target velocity, and the gray line is the absolute error, defined as the target velocity minus the predicted v.

**Figure 46:**
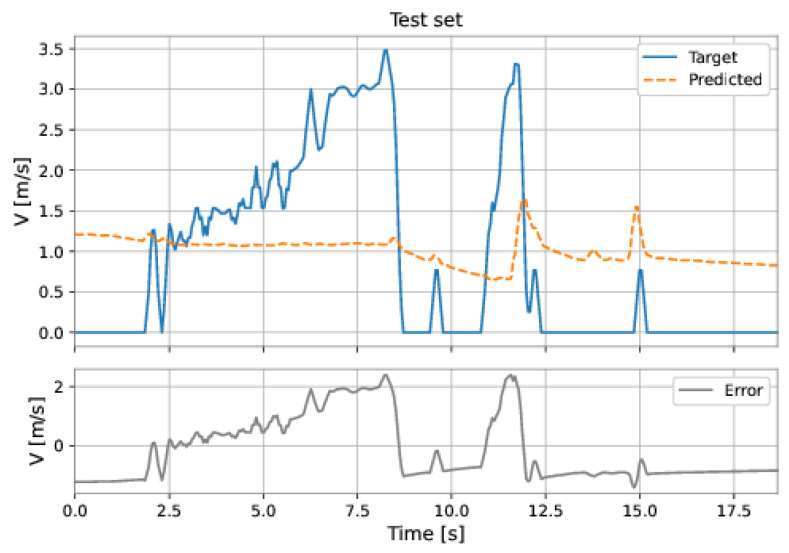
Linear regression predictions on the test sets. The orange dashed line is the velocity predicted by the model, the blue solid line is the target velocity, and the gray line is the absolute error, defined as the target velocity minus the predicted v.

Figure 45 and Figure 46 show the predictions of Model 3 (Table 5) for the training and test sets. This model presented the best performance (smaller RMSE) on the test set. The poor performance of the model confirms that it is not suitable to predict the PIG’s velocity in our datasets. Next, we present different neural networks developed to predict the PIG’s velocity.

For all the following networks, the statements below apply:

- The parameters of the network (synaptic weights) were adjusted with the adaptive moment estimation (Adam) algorithm, a gradient-based optimization algorithm [37]. The loss function was the mean squared error (MSE);
- The optimizer’s learning rate and the model’s hyperparameters were automatically chosen with a random search using the KerasTuner library. The search space was described for each model;
- We configured KerasTuner to randomly select 50 combinations of the hyperparameters comprised on the search space. For each combination of hyperparameters, the model was fitted three times;
- The activation function of the hidden layers was the rectified linear unit (ReLU), and the activation function of the output layer was the linear function;
- Aiming to avoid overfitting, we applied the dropout technique (rate = 20%) in the hidden layers of the MLP models;
- We used a technique known as early stopping to define the number of epochs (iterations) over which the network was trained.

#### 4.3.1. Multilayer Perceptron (MLP)

We built a multilayer perceptron (MLP) to predict the velocity using different combinations of features (pressures and accelerations). The search space for the MLP model was defined as follows:

- Number of layers: {1,2,3,4,5};
- Number of neurons in each hidden layer: {16,32,48,64,⋯,256};
- Learning rate: {0.01,0.001,0.0001}.

**Figure 47:**
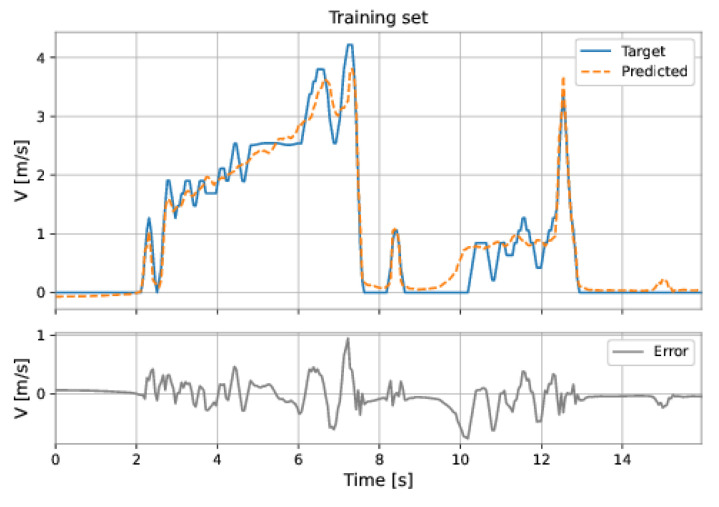
MLP’s predictions on the training set.

**Figure 48:**
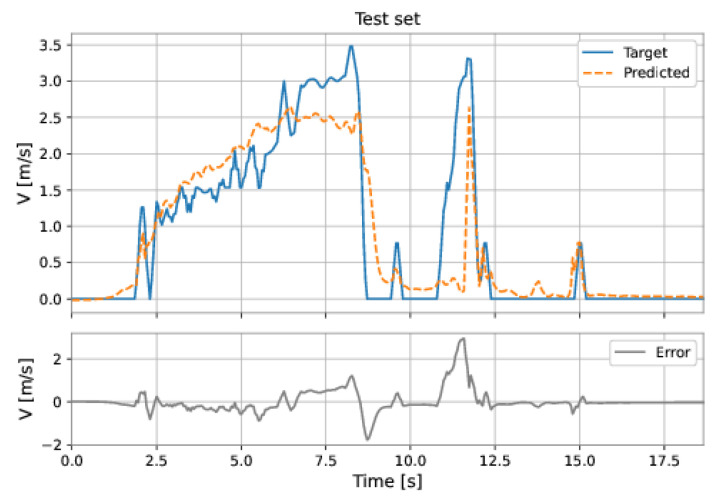
MLP’s predictions on the test set.

The MLP network used all the features (pressure upstream, pressure downstream, differential pressure, accelerations on the three axes, and total acceleration). The model has two hidden layers: 224 neurons in the first hidden layer and 224 neurons in the second hidden layer; the learning rate was equal to 0.001. The root mean square error (RMSE) on the training set was 0.2217 m/s and on the test set was 0.5457 m/s. Figure 47 and Figure 48 show the results obtained by the model on the training and test sets.

#### 4.3.2. MLP-TDNN

The network referred to as MLP-TDNN is a time-delay neural network whose inputs are the pressures and accelerations at the current instant and past instants. We tried different combinations of inputs and orders of delay (from 1 to 6). The search space for the random search was defined as follows:

- Number of layers: {2,3,4,5,6,7};
- Number of neurons in each hidden layer: {16,32,48,64,⋯,256};
- Learning rate: {0.01,0.001,0.0001,0.00001}.

The MLP-TDNN model used all the features and a delay of order 1 in the inputs. The model presents three hidden layers: 64 neurons in the first hidden layer, 80 neurons in the second hidden layer, and 80 neurons in the third hidden layer; the learning rate was equal to 0.001. The RMSE on the training set was 0.2548 m/s and on the test set was 0.6091 m/s.

**Figure 49:**
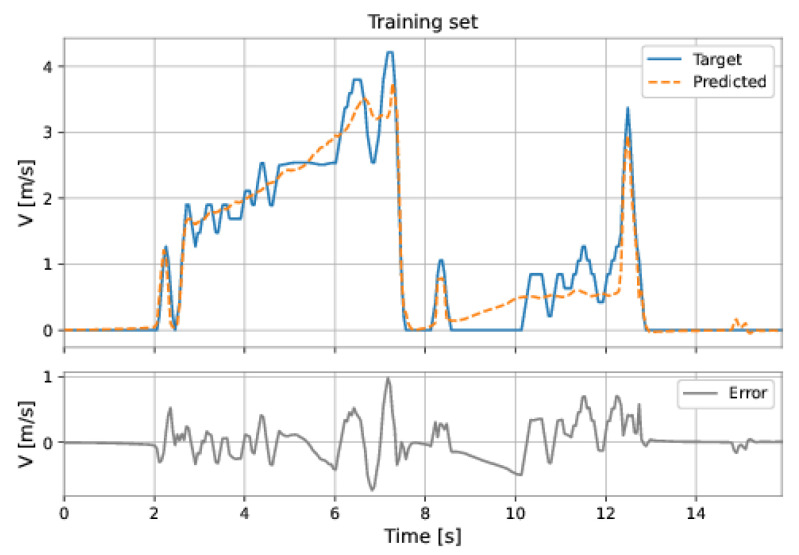
MLP-TDNN’s predictions on the training set.

**Figure 50:**
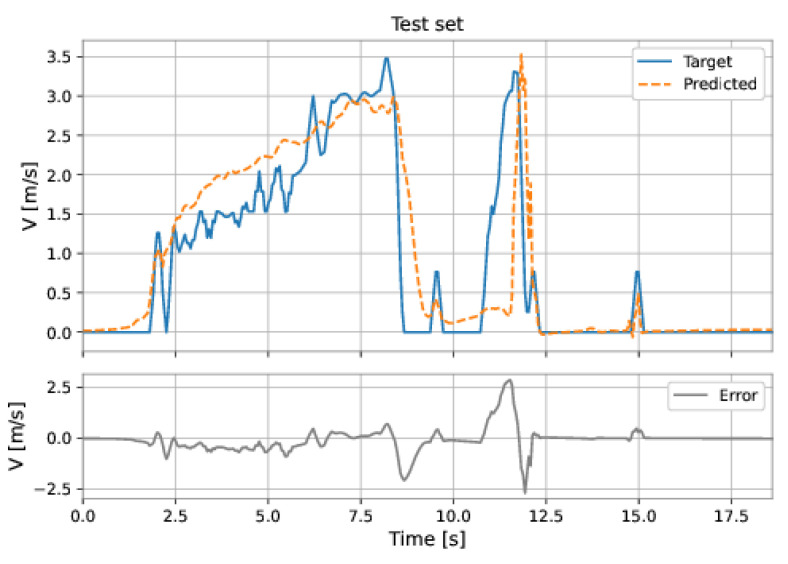
MLP-TDNN’s predictions on the test set.

Figure 49 and Figure 50 show the results obtained by the model on the training and test sets.

#### 4.3.3. LSTM-TDNN

Analogous to the MLP-TDNN from the last section, the LSTM-TDNN is a long short-term memory network whose inputs are the pressures and accelerations at the current instant and previous instants. In this case, the architecture of the LSTM-TDNN is made up of a single LSTM layer stacked with an MLP network. The search space was defined as follows:

- Number of neurons of the LSTM network: {10,20,30,40,50};
- Number of layers of the MLP network: {2,3,4,5,6,7};
- Number of neurons in each hidden layer of the MLP: {16,32,48,64,⋯,256};
- Learning rate: {0.01,0.001,0.0001,0.00001}.

**Figure 51:**
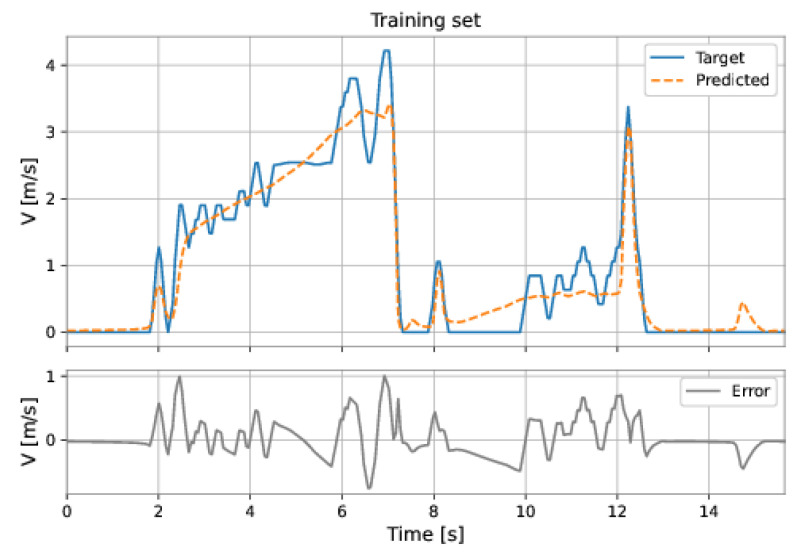
MLP’s predictions on the training set.

**Figure 52:**
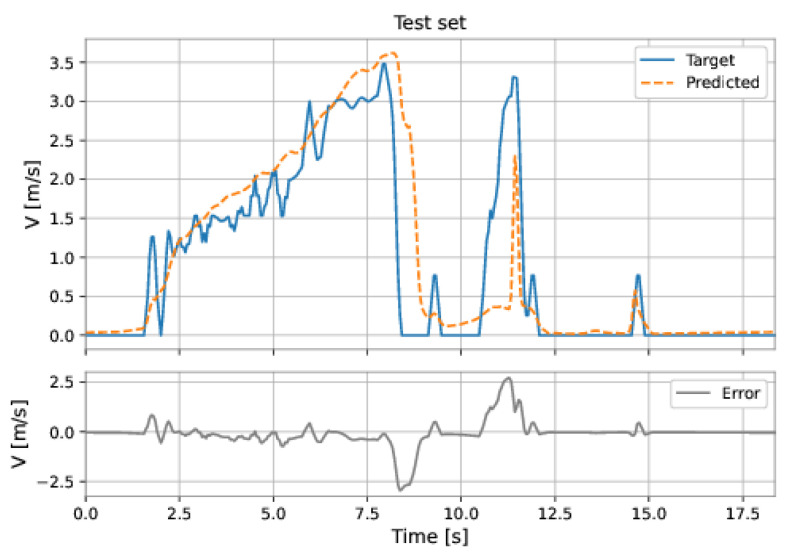
MLP’s predictions on the test set.

The LSTM-TDNN used all the features and a delay of order 6 in the inputs. The model presents an LSTM layer with 50 neurons stacked with an MLP network with three hidden layers: 224 neurons in the first hidden layer, 160 neurons in the second hidden layer, and 160 neurons in the third hidden layer; the learning rate was equal to 0.001. The RMSE on the training set was 0.2875 m/s and on the test set was 0.6591 m/s. Figure 51 and Figure 52 show the results obtained by the model on the training and test sets.

#### 4.3.4. MLP-NARX

The model referred to as MLP-NARX is a non-linear autoregressive network with an exogenous inputs network. It refers to the series-parallel (open-loop) operation when the model makes a one-step prediction; given the current input, the past inputs, and the past true outputs, the model predicted the current output. Again, we tried different combinations of inputs and different orders of delay (from 1 to 6). However, in this model, the delays were applied to both the inputs and the feedback output (velocity). The search space for the random search was defined as follows:

- Number of layers: {2,3,4,5,6,7};
- Number of neurons in each hidden layer: {16,32,48,64,⋯,256};
- Learning rate: {0.01,0.001,0.0001}.

**Figure 53:**
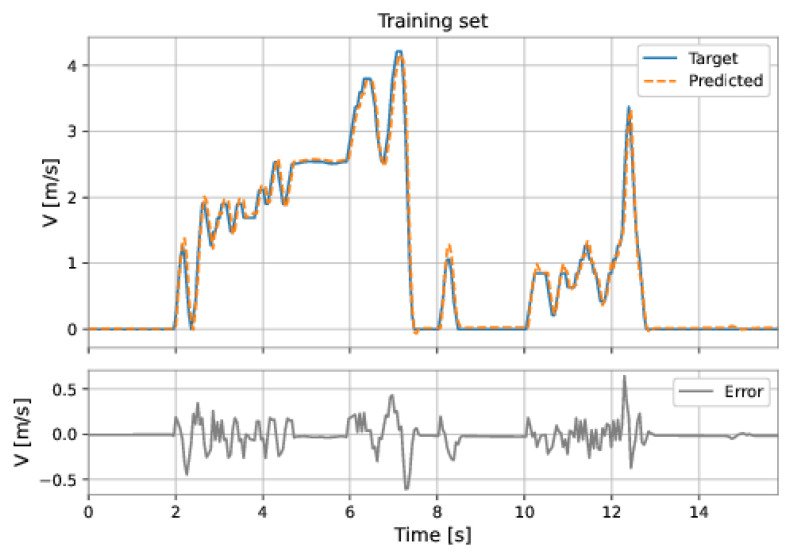
MLP’s predictions on the training set.

**Figure 54:**
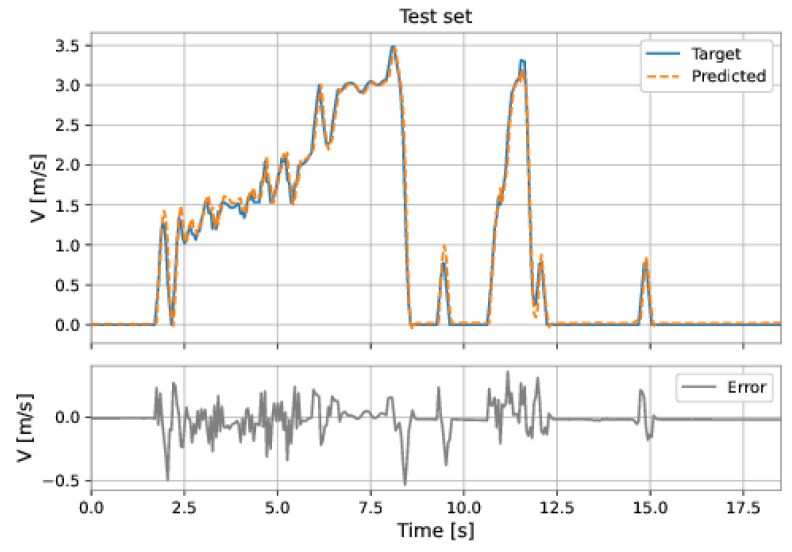
MLP’s predictions on the test set.

The MLP-NARX model’s features were the differential pressure and total acceleration on the current instant and past instants, as well as the feedback velocity on previous instants. The order of input delay is 1 and the order of output delay is 3. The model presents two hidden layers: 160 neurons in the first hidden layer and 192 neurons in the second hidden layer; the learning rate was equal to 0.001. The RMSE on the training set was 0.1314 m/s and on the test set was 0.1057 m/s. Figure 53 and Figure 54 show the results obtained by the model on the training and test sets.

#### 4.3.5. LSTM-NARX

Analogous to the MLP-NARX, the LSTM-NARX is a long short-term memory network whose inputs consist of current inputs, past inputs, and feedback from past outputs. Similarly, the LSTM-NARX refers to the series-parallel (open-loop) operation. The model’s architecture is made up of a single LSTM layer stacked with an MLP network. The search space was defined as follows:

- Number of neurons of the LSTM network: {10,20,30,40,50};
- Number of layers of the MLP network: {2,3,4,5,6,7};
- Number of neurons in each hidden layer of the MLP: {16,32,48,64,⋯,256};
- Learning rate: {0.01,0.001,0.0001,0.00001}.

**Figure 55:**
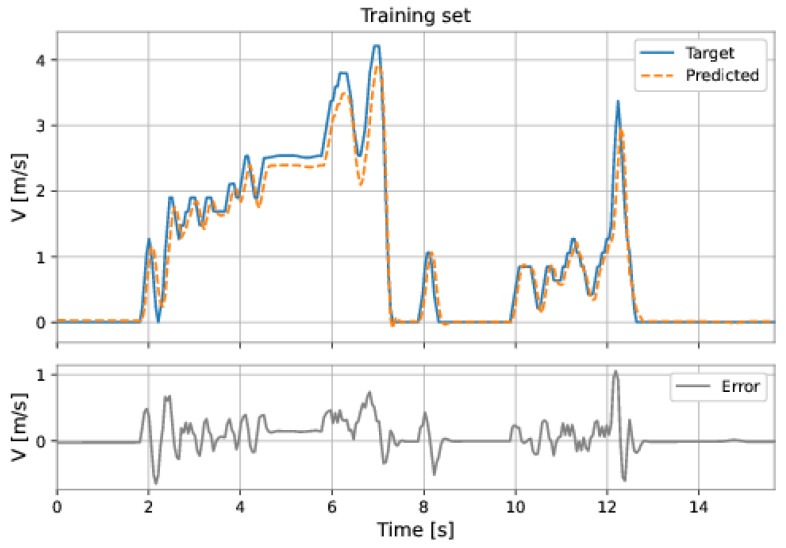
LSTM-NARX results (series-parallel). Training set.

**Figure 56:**
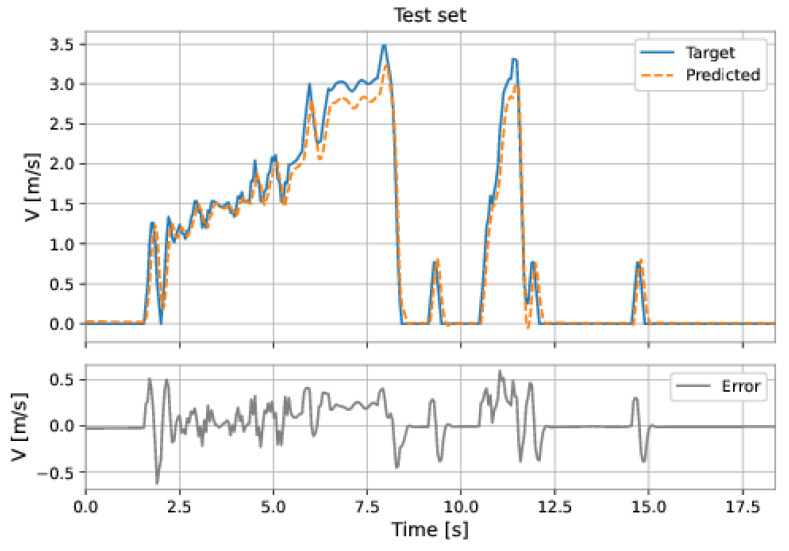
LSTM-NARX results (series-parallel). Test set.

The LSTM-NARX model used the differential pressure and total acceleration on the current instant and past instants, as well as the fed-back velocity on previous instants. The order of input delay is 1 and of the output delay, is 6. The model presents an LSTM layer with 25 neurons stacked with an MLP network with three hidden layers: 96 neurons in the first hidden layer, 48 neurons in the second hidden layer, and 64 neurons in the third hidden layer; the learning rate was equal to 0.001. The RMSE on the training set was 0.2248 m/s and on the test set was 0.1780 m/s. Figure 55 and Figure 56 show the results obtained by the model on the training and test sets.

### 4.4. Summary of Results

Table 6 summarizes the models’ performances, presenting the root mean square error (RMSE) obtained by each model on the training and test sets.

We used different neural network architectures and different combinations of input variables to search for models with reasonable prediction performance. THE MLP-NARX and LSTM-NARX showed the best performances. However, it is worth noting that these results refer to the serial-parallel operation of the models, requiring the true past outputs to predict the current output, while MLP required only the current inputs. Although MLP does not include an explicit framework for temporal pattern processing, it performed better than models such as MLP-TDNN and LSTM-TDNN on our datasets.

### 4.5. Discussion

We found artificial neural networks can predict the velocity of a pipeline inspection gauge (PIG) using the differential pressure that acts on the device. This finding agreed with its dynamical model (Section 3), which stated that differential pressure is its driving force. In addition, the PIG’s acceleration, as measured by the accelerometer, was demonstrated to enhance the performance of the networks. This was also expected since acceleration correlates with velocity.

We trained several neural networks with experimental data collected during two runs of a prototype PIG in a testing pipeline. We used the data collected on the first run to train the model, then used the data from the latter to evaluate it. The results show that the models developed can predict the velocity with acceptable performance even on previously unseen data (test set). Additional data are required, though, to verify the generalization capability of the models and to select the best model among those already developed.

A simpler and widely applied technique for obtaining the velocity of PIGs is the employment of odometers. Their drawback is that they exhibit significant measurement errors mainly related to the slipping and contact loss between the odometer wheel and the pipeline’s wall.

A basic approach for reducing these errors is to modify the constructive elements of the odometer, such as the springs and the wheel surface, and to alter the friction force between the wheel and the pipe wall. However, as often happens with mechanical devices, the odometer is prone to fail [10]. Other investigations have employed the odometer as the primary sensor but included additional information to compensate for its measurement issues. In [38], for example, the location of welds inside the pipeline was used; in [8], combined data from an odometer, a low-cost inertial measurement unit (IMU), and topographic landmarks were used; and in [10], an IMU was used with the location of pipeline junctions. Finally, [12] used the differential pressure that acts on the PIG inside the pipeline to predict its velocity. These investigations share a common characteristic: they each estimated the velocity after the PIG’s retrieval from the pipeline (offline).

Similarly, [12] employed neural networks to predict the PIG’s velocity. In contrast, we developed a prototype PIG with embedded sensors rather than requiring data from external pressure sensors that are only available upon PIG retrieval. Thus, our system can measure the differential pressure and, therefore, predict the PIG’s velocity during its run inside the pipeline (online). The main implication of online prediction is that it allows the system to be used by a velocity controller embedded in the PIG.

Finally, this study represents a valuable contribution to the velocity measurement of pipeline inspection gauges (PIGs); the oil and gas industry can benefit from our results to improve the quality of maintenance operations with PIGs, using the velocity prediction model as a complement to odometer-based techniques.

## 5. Conclusions

A model was developed for predicting the PIG velocity based on the differential pressure that acts on the PIG inside the pipeline. The main motivation was to provide an alternative to velocity measurement methods based on the use of odometers, as they produce significant measurement errors, mainly caused by the loss of contact between the odometer wheel and the duct surface. The system proposed in this paper differs fundamentally from previous approaches in terms of applicability since online measurement enables the use of an estimator for the application of PIG velocity control. Therefore, we anticipate that our findings will contribute to improving the functioning of velocity controllers for PIGs and, consequently, to increasing the efficiency of maintenance operations in the pipeline system. In future investigations, the performance of non-linear regression models should be considered.

## Figures and Tables

**Table 1:** Main features of the Raspberry Pi used in this investigation (Raspberry Pi 3 Model B+).

Parameter	Value
Operating system	Raspbian GNU/Linux 10 (buster)
Processor	Cortex-A53 (ARMv8) 64 bits quad-core
Clock	1.4 GHz
RAM memory	1 GB
I/O interface	40 GPIO pins
Communication	Bluetooth 4.2, IEEE 802.11 5 GHz, Gigabit Ethernet
Dimensions	85 × 56 × 17 mm

**Table 2:** Features of the pressure sensors.

Features	Description
Working principle	Hall effect
Pressure range	0–5 bar
Output voltage	0.5–4.5 VDC
Supply voltage	5 VDC
Response time	2.0 ms
Measurement accuracy	±1.5% FS (75 mbar)

**Table 3:** Pressures and corresponding voltages for the pressure sensors.

Pressure (bar)	Upstream Sensor (V)	Downstream Sensor (V)
0.0	0.504	0.511
1.0	0.996	1.00
2.0	1.803	1.805
3.0	2.711	2.719
4.0	3.497	3.503
5.0	4.424	4.427

**Table 4:** Main features of the embedded system’s power bank.

Feature	Description
Battery type	Lithium Polymer (LiPo)
Capacity	5000 mAh
Output voltage	5 VDC
Output current	2 A

**Table 5:** Performance of the linear regression models. Each model used a different combination of features. All means that the model used all the features from the data sets.

Model	Features	Training	Test
1	All	1.1118	1.2765
2	ΔP, $acc_{x}$ acc_total	1.1498	1.0874
3	ΔP, acc_total	1.1504	1.089
4	$P_{up}$, $P_{down}$, ΔP, acc_total	1.1186	1.1186

**Table 6:** The root mean square error (RMSE) on the training and test sets obtained by each model.

Model	Training	Test
MLP	0.2217	0.5457
MLP-TDNN	0.2548	0.6091
LSTM-TDNN	0.2875	0.6591
MLP-NARX	0.1314	0.1057
LSTM-NARX	0.2248	0.1780

## Abbreviations

The following abbreviations are used in this manuscript:

PIG	Pipeline inspection gauge
EKF	Extended Kalman filter
IMU	Inertial measurement unit
MEMS	Micro-electromechanical system
ANN	Artificial neural network
ILI	In-line inspection
LSTM	Long short-term memory
TDNN	Time-delay neural network
NARX	Non-linear autoregressive network with exogenous inputs
ADC	Analog-to-digital converter
SPI	Serial peripheral interface
SBC	Single-board computer
IoT	Internet of Things
CSV	Comma-separated values
SSH	Secure shell
RMSE	Root mean square error
ADAM	Adaptive moment estimation
MSE	Mean squared error
ReLU	Rectified linear unit
MLP	Multilayer perceptron

## References

[1] Nguyen T.T., Kim S.B., Yoo H.R., Rho Y.W. (2001). Modeling and simulation for PIG flow control in natural gas Pipeline. KSME Int. J..

[2] Nguyen T.T., Yoo H.R., Rho Y.W., Kim S.B. Speed control of pig using bypass flow in natural gas pipeline. Proceedings of the 2001 IEEE International Symposium on Industrial Electronics Proceedings, ISIE 2001.

[3] Yardi C.N. (2004). Design of Regulated Velocity Flow Assurance Device for Petroleum Industry. Master’s Thesis.

[4] Haniffa M.A., Hashim F.M. (2012). Recent developments in speed control system of pipeline pigs for deepwater pipeline applications. World Acad. Sci. Eng. Technol. J. Mech. Mechatronics Eng..

[5] Liang Z., He H., Cai W. (2017). Speed simulation of bypass hole PIG with a brake unit in liquid pipe. J. Nat. Gas Sci. Eng..

[6] Sun H., Peng L., Huang S., Li S., Long Y., Wang S., Zhao W. (2022). Development of a Physics-Informed Doubly Fed Cross-Residual Deep Neural Network for High-Precision Magnetic Flux Leakage Defect Size Estimation. IEEE Trans. Ind. Informatics.

[7] Lu S., Feng J., Zhang H., Liu J., Wu Z. (2019). An Estimation Method of Defect Size From MFL Image Using Visual Transformation Convolutional Neural Network. IEEE Trans. Ind. Informatics.

[8] Santana D.D.S., Maruyama N., Furukawa C.M. Estimation of trajectories of pipeline PIGs using inertial measurements and non linear sensor fusion. Proceedings of the 2010 9th IEEE/IAS International Conference on Industry Applications (INDUSCON 2010).

[9] Money N., Cockfield D., Mayo S., Smith G. (2016). Dynamic speed control in high velocity pipelines. Pipeline Gas J..

[10] Zhu X., Li X., Zhao C., Zhang S., Liu S. (2016). Dynamic simulation and experimental research on the motion of odometer passing over the weld. J. Nat. Gas Sci. Eng..

[11] Sahli H., El-Sheimy N. (2016). A Novel Method to Enhance Pipeline Trajectory Determination Using Pipeline Junctions. Sensors.

[12] De Araújo R.P., De Freitas V.C.G., De Lima G.F., Salazar A.O., Dória Neto A.D., Maitelli A.L. (2018). Pipeline Inspection Gauge’s Velocity Simulation Based on Pressure Differential Using Artificial Neural Networks. Sensors.

[13] Zhu X., Zhao C., Li X., Zhang S., Liu S. (2019). Direct observation of odometer trajectory when passing over weld in oil and gas pipeline. J. Pipeline Syst. Eng. Pract..

[14] Narendra K.S., Parthasarathy K. (1990). Identification and control of dynamical systems using neural networks. IEEE Trans. Neural Networks.

[15] Sjöberg J., Hjalmarsson H., Ljung L. (1994). Neural Networks in System Identification. IFAC Proc. Vol..

[16] Sjöberg J. (1995). Non-Linear System Identification with Neural Networks.

[17] Habtom R. Soft-sensing using recurrent neural networks. Proceedings of the 1998 IEEE International Symposium on Intelligent Control (ISIC) held jointly with IEEE International Symposium on Computational Intelligence in Robotics and Automation (CIRA).

[18] Haykin S. (2000). Neural Networks: Principles and Practice.

[19] Ferrari S., Piuri V., Ablameyko S., Goras L., Piuri M.G.V. (2003). Introduction to neural networks for instrumentation, measurement, and industrial applications. Neural Networks in Intelligent Sensors and Measurement Systems for Industrial Applications.

[20] Fortuna L., Graziani S., Rizzo A., Xibilia M.G. (2007). Soft Sensors for Monitoring and Control of Industrial Processes.

[21] Abiodun O.I., Jantan A., Omolara A.E., Dada K.V., Mohamed N.A., Arshad H. (2018). State-of-the-art in artificial neural network applications: A survey. Heliyon.

[22] Lima G.F., Freitas V.C.G., Araújo R.P., Maitelli A.L., Andrés O., Salazar A.O. (2017). Pig’s speed estimated with pressure transducers and hall effect sensor: An industrial application of sensors to validate a testing laboratory. Sensors.

[23] Freitas V.C., Lima G.F., Salazar A.O., Maitelli A.L. (2016). “PIG” Detection with Pressure Transducers. J. Adv. Res. Electr. Electron. Instrum. Eng..

[24] Nieckele A.O., Braga A.M.B., Azevedo L.F.A. (2001). Transient Pig Motion Through Gas and Liquid Pipelines. ASME. J. Energy Resour. Technol..

[25] Chollet F. (2017). Deep Learning with Python.

[26] Mitchell T.M. (1997). Machine Learning.

[27] Silva I.N., Spatti D.H., Flauzino R.A. (2016). Redes Neurais Artificiais para Engenharia e Ciências Aplicadas.

[28] Aguirre L.A. (2015). Introduction to Systems Identification.

[29] Tangirala A.K. (2015). Principles of System Identification: Theory and Practice.

[30] Aguirre L.A. (2007). Enciclopédia de Automática: Controle e Automação.

[31] Hochreiter S., Schmidhuber J. (1997). Long Short-Term Memory. Neural Comput..

[32] Liu S., Zheng D., Li R. (2019). Compensation Method for Pipeline Centerline Measurement of in-Line Inspection during Odometer Slips Based on Multi-Sensor Fusion and LSTM Network. Sensors.

[33] Barnatt C. Single Board Computers. Raspberry Pi Foundation. https://www.explainingcomputers.com/sbc.html.

[34] Lima G.F. (2019). Speed Control Proposal for Tool Inspection Inspection (PIG) Using Bypass Valve. Ph.D. Thesis.

[35] VectorNav, Inertial Navigation Primer. Learn about Mems Accelerometers, Gyroscopes, and Magnetometers VectorNav. https://www.vectornav.com/resources/inertial-navigation-primer/theory-ofoperation/theory-mems.

[36] Pedregosa F., Varoquaux A.G., Gramfort V., Michel B., Thirion O., Grisel M., Blondel P., Prettenhofer R., Weiss V., Dubourg J. (2011). Scikit-learn: Machine learning in Python. J. Mach. Learn. Res..

[37] Kingma D.P., Ba J. (2015). Adam: A method for stochastic optimization. arXiv.

[38] Sadovnychiy S., López J., Ponomaryov V., Sadovnychyy A. (2006). Evaluation of distance measurement accuracy by odometer for pipelines pigs. J. Jpn. Pet. Inst..

